# Suppression of mycotoxins production and efficient chelation of heavy metals using natural melanin originated from *Aspergillus flavus* and *Aspergillus carbonarius*

**DOI:** 10.1186/s12896-024-00941-7

**Published:** 2025-01-11

**Authors:** Nashwa El-Gazzar, Esraa Abdo, Gamal Rabie, Manal Tawfeek El-Sayed

**Affiliations:** Department of Botany and Microbiology, Faculty of Science, Zagazig University, Zagazig, Sharkia 44519 Egypt

**Keywords:** Fungal metabolites, Natural melanin pigment, Antioxidant activity, Aflatoxin-B1, Ochratoxin-A, Heavy metal chelation, Bioremediation, Molecular docking

## Abstract

**Background:**

This study employed melanin synthesized by *Aspergillus flavus* and *Aspergillus carbonarius* to inhibit the production of mycotoxins and bioremediation of heavy metals (HMs).

**Methods:**

First, twenty fungal isolates were obtained from soil samples, and were evaluated to produce melanin. The melanin of the most potent producers has undergone several confirmatory experiments, including, Dihydroxyphenylalanine (DOPA)-inhibitor-kojic acid pathway detection, High-performance liquid chromatography (HPLC), Fourier-transform infrared (FTIR) and Nuclear magnetic resonance (NMR). Additionally, the melanin production culture conditions were optimized. The antioxidant activity of melanin was detected with 1,1-Diphenyl-2-picrylhydrazyl (DPPH). HPLC was used to measure the mycotoxins produced in culture media supplemented with melanin. Molecular docking study investigated molecular interactions between melanin and mycotoxins through *in silico* approaches. FTIR and Energy-dispersive X-ray spectroscopy (EDX) were utilized to determine the percentage of melanin-chelated HMs, and an atomic absorption spectrophotometer (AAS) was used to detect HMs removal efficiency.

**Results:**

The melanin-enriched medium (0.3% and 0.4%) exhibited complete inhibition of aflatoxin B1 (AF-B1) by *A. flavus* and ochratoxin A (OTA) by *A. carbonarius*, respectively. Furthermore, melanin showed effective HM removal efficiency, increasing with melanin concentration. The removal efficiency of Cd^+2^ and Cr^+6^ by 1 mg/mL melanin was 49% and 63%, respectively. When the concentration of melanin was increased to 15 mg/mL, the removal efficiency of Cd^+2^ and Cr^+2^ increased to 60% and 77%, respectively.

**Conclusion:**

The study exhibited a natural approach for melanin production, using melanin as a heavy metal-chelating agent and capability to inhibit the production of aflatoxin B1 and ochratoxin A. Further, the study provides significant evidence regarding the bioremediation pipeline, for melanin production through biotechnological processes by filamentous fungi.

**Supplementary information:**

The online version contains supplementary material available at 10.1186/s12896-024-00941-7.

## Background

Heavy metals (HMs) and mold contamination pose significant risks to human health and the environment due to their toxicity, persistent presence, and capacity for bioaccumulation [1–3]. Among the most hazardous HMs are chromium [Cr], cadmium [Cd], lead [Pb], and mercury [Hg], as they tend to accumulate in multiple organs [4, 5]. Consequently, mycotoxin and HM toxicity is a global health concern, necessitating further research to discover novel approaches to combat the harmful effects of these toxins as well as non-conventional protocols.

Numerous remediation techniques, including chemical precipitation, photocatalytic adsorption, destruction, and filtration, have been previously developed [6, 7]. The global popularity of safe materials for therapeutic purposes and biosorptive agents for hazardous environmental pollutants is steadily increasing [8]. In this framework, natural products offer a promising prospect for effectively inhibiting mycotoxin release and bioremediation of heavy metal toxicity [8–10]. The low cost and simple adsorption procedure are viable, particularly for non-degradable HMs [11]. Hence, melanin is a natural agent with outstanding heavy metal removal properties [12].

Melanin is a dark, high molecular-weight pigment synthesized through hydroxylation and polymerization of organic compounds [13–15]. It is a potent ion exchange molecule that effectively chelates pollutants, chemicals, and HMs [16]. In addition, the capacity of melanin to chelate compounds and control their cellular uptake, and thus, it functions as a trap or storage site for metal ions [17, 18]. Further, melanin was found to possess mycotoxin detoxification properties [10]. Despite these remarkable characteristics of melanin, it is still challenging to produce it on a large scale since the chemical synthesis is expensive and environmentally unfriendly [19]. Evidently, melanin production from plants and animals has some limitations imposed by the natural growth cycle and environment [20].

Microbial melanin is found in the cell wall and produced through enzymatic reactions involving tyrosinase and polyketidesynthase from DOPA and dihydroxynaphthalene (DHN), respectively [21]. Currently, the researches focus on highlighting newly natural melanin-inducing progeny of filamentous fungi through fermentation technology [14, 22, 23]. Therefore, the current study was designed to produce and refine a melanin pigment using *A. flavus* and *A. carbonarius* in a submerged culture. The melanin was characterized using various techniques, including physical and chemical analysis, UV, HPLC, FT–IR, and NMR. Moreover, their antioxidant activity, suppression of AF–B1 and OTA production, and removal of HMs were studied.

## Materials and methods

### Sample collection, isolation, and purification of fungi

A total of 12 rhizosphere soil samples were collected from various regions in Sharkia Governorate, Egypt. The soil samples were collected and transferred to the lab on the same day for the isolation of fungi. The serial dilution plating method was used to dilute the soil samples to minimize the number of fungal colonies in each soil dilution [24]. The stock soil solution was prepared by dissolving 50 g of each soil sample (separately) in 100 mL 85% NaCl, with thorough agitation. The solution was then diluted into a series of prepared vials labeled from 10–1 to 10–6, each containing 9 mL 85% NaCl. One milliliter of the soil stock solution was transferred to the first vial. Subsequently, another 1 mL of the solution from the first vial was transferred to the second vial, and the steps were repeated until the last dilution. Czapek Dox Agar (CDA) (Hi Media Lab. Pvt. Ltd. Mumbai, India; Ref. GM075) plates were prepared, and 0.1 mL of each dilution was pipetted and spread on the prepared CDA plates. The plates were incubated at 28–30℃ for 5–7 days. In order to obtain pure fungal isolates, the appeared colonies were then sub-cultured on sterile CDA plates and incubated for 5–7 days at 28–30℃.

### Morphological and molecular identification

The obtained fungal pure isolates were identified based on their macroscopic and microscopic morphological characteristics [25–27]. These isolates were further screened for their potential to produce melanin, and the most potent producers were confirmed by molecular identification of the 18–28S rRNA gene at SolGent Company in Daejeon, South Korea. The forward and reverse primers ITS1 (5′-TCCGTAGGTGAACCTGCGG-3′) and ITS4 (5’-TCCTCCGCT TATTGATATGC-3′) [28]. were used for PCR amplification of 18-28S rRNA gene. The purified PCR amplicons were sequenced using the same primers. The obtained sequences were further searched for similarities with nucleotide sequences in the Gen-Bank using the BLAST (BLASTn) search tool, and sequences were deposited in the Gen-Bank for accession number. The MegAlign (DNA Star) software version 5.05 (DNASTAR Inc., Madison, WI, USA) [#] [DNASTAR Inc. (2003) [29], DNASTAR software, version 5.05 was used to conduct a phylogenetic analysis of the sequences.

### Screening of melanin production

The twenty fungal isolates were screened for their potential to produce melanin. The fungal isolates were inoculated separately in Czapek Dox Liquid Medium (Hi Media Lab. Pvt. Ltd., India, Ref. M1170A) and incubated at 30℃ for seven days. Then, the cultures were filtered, and the mycelia were used to extract melanin. As described by El-Sayyad et al. [30, 31], approximately 1 g of mycelial biomass of each isolate was sterilized with 1 N NaOH for 20 min at 121 °C and 1.5 bar. Subsequently, the mixture was centrifuged at room temperature for 10 min at 8000 rpm, and the supernatant, including melanin, was gathered. HCL with pH 2 was used for condensing the melanin pigment at 10,000 rpm for 10 min at 4℃. Afterward, the pigment was washed thrice with ethyl acetate; chloroform (2;3, v/v) and then centrifuged three times with distilled water. The gathered melanin was cut in dehumidified air. Lastly, the pigments were dissolved in a 1 M KOH to a final concentration of 0.01 µg/mL and analyzed via UV-Vis (T60 UV/Vis. 200–900 nm) with a scanning interval of 1 nm, where L-DOPA (PHR1271-500MG, Sigma USA) served as a standard. The biomass dry weight of melanin was estimated as described by Kumar et al. [32], El-Batal & Al Tamie [33], Joshi et al. [34].

### Physicochemical characterization and pathway mechanism for melanin production

The extracted melanin pigments from *A. flavus* and *A. carbonarius* were identified based on their physical and chemical properties, including solubility in water, color observation, solubility in KOH, precipitation in 3N HCL, solubility in organic acid solvent (methanol, ethanol, hexane, chloroform, benzene, acetone, and ethyl acetate), reaction with H_2_O_2_, reaction with FeCl_3_, (reaction for polyphenols test), and reaction with sodium dithionite and potassium ferricyanide [22, 35].

The melanin passage mechanism was elucidated by assessing the impact of inhibitors, such as Kojic acid which suppresses DOPA, and tricyclazole, which inhibits DHN. Kojic acid was solubilized in distilled H_2_O, while tricyclazole was dissolved in ethanol. They were subjected to an autoclaved and cooled PDA medium to denote rates of 1, 10, or 100 µg/mL [36]. The cultures were acclimated for seven days at 25 °C, after which the pigmentation and growth were observed [14, 22].

### HPLC analysis

High-performance liquid chromatography (HPLC) analysis was used to identify the precursor molecules and intermediates involved in melanin synthesis. The purified melanin was anatomized by HPLC (Thermo scientific HPLC system, Santa Clara, USA) using C18 column (Eclipse Plus C18 4.6_150 mm, 3.5_m, Cat.# 959963-902) through isocratic mobile stage of methanol and 1% acetic acid with a flux average of 1.0 mL/min for 20 min. Prior to injection onto the column, the specimen was filtered using a 0.2 µm filter (Millipore, Amicon, Mumbai, India). The analysis concentrated on the adherence to chemical standards and the process of condensation that occurred during the detention period. This was accomplished through the diligent effort of the assimilation summit. The melanin was amplified using L-DOPA (PHR1271-500MG, Sigma USA) as a standard [37].

### FTIR and NMR spectral analysis

Fourier-transform infrared (FTIR) spectral analysis and Nuclear Magnetic Resonance (NMR) spectroscopy of the purified fungal melanin were conducted at a micro-analytical lab at Cairo University, Egypt. The FTIR spectral analysis was performed at room temperature with disbanding whole reflection by an FTIR (Perkin Elmer). The specimens were fused and compressed with KBr, then maintained at a rate of 650–4000 cm^−1^ [34, 38, 39]. Output signs have been described and analyzed using spectra software.

For NMR spectroscopy of melanin, approximately 20 mg of purified melanin was mixed with 1 mL of reiterated dimethyl sulfoxide, filtered through a small cotton sleeve tightly packed into a Pasteur pipette, and transferred to the proton NMR device (^1^H NMR). The purified sample was analyzed in sol ECA-500 with cry-probe operating at 500 MHz with DMSO at 205 ppm. The specimens were settled by solubility of 50 mg from U7 melanin and the standard in 3.5 mL of deuterium oxide/ammonia combination. The mixture was made by mixing 0.01 mL of hydrous ammonia (33%) with 10 mL of deuterium oxide at pH 10 [40, 41].

### Optimization of melanin production conditions

To determine the optimal conditions for the production of melanin by the most potent strains, different media, incubation temperature, incubation period, pH levels, carbon sources, nitrogen sources, and heavy metals were assessed [42–44]. During the optimization experiments, UV-Vis analysis was utilized to measure the concentration of melanin, using L-DOPA as a reference standard. Additionally, the dry weight was estimated in each experiment. Potato dextrose broth medium (PD), Mineral salt medium (MS), and Czapek–Dox broth medium (CD) were used to test their effect on the production of melanin by *A. flavus* and *A. carbonarius*. Three sterilized replicates of each medium (pH 5.5) were inoculated with 1 mL of spore suspension 2.0 × 10^6^ spores/mL. Cultures were then incubated at 30 °C for seven days. Subsequently, the melanin production was evaluated as described in the previous step. After the determination of the optimal medium for melanin production, the medium was adjusted at varying pH levels ranging from pH 2 to pH 8. Afterward, the cultures with pH adjustments were subjected to incubation at various temperatures, ranging from 20 to 40℃. The optimal incubation period was determined in the following manner. The fungal cultures were incubated for a total period of 16 days with Melanin production detected on days 3, 5, 7, 10, 12, 14, and 16.

In order to determine the best carbon source for melanin production, the main carbon source in the CD medium was replaced with each of the tested carbon sources at the same concentration as the original carbon source. Therefore, the CD medium, which did not contain the original carbon source, was supplemented with 3% (the concentration of the original carbon source in the CD medium) of soluble starch, glucose, lactose, fructose, maltose, and pectin separately. Subsequently, the carbon source with the highest quality was analyzed at various concentrations to determine the optimal concentration. Similarly, to determine the most effective nitrogen source for enhancing melanin production, the original nitrogen source of the CD medium was replaced with the tested nitrogen sources (each tested separately) at the original nitrogen concentration of the medium. Consequently, the following nitrogen sources were added to the medium in dissimilar amounts: NaNO_3_ (3 g/100 mL), (NH_4_)_2_ SO_4_ (2.33 g/100 mL), urea (1.12 g/100 mL), yeast extract (3.33 g/100 mL), NH_4_H_2_PO_4_ (4.2 g/100 mL), beef extract (1.6 g/100 mL) and peptone (2.19 g/100 mL). Subsequently, the nitrogen source promoting melanin production at the optimal level was tested at different quantities to detect the optimal concentration. After setting up the optimal production medium, pH, incubation temperature, incubation period, carbon source, and nitrogen source, the production of melanin in the presence of 0.1, 0.2, 0.5, 1, 2, 3, and 5 mM of different heavy metal sources (CuSO_4_.5H_2_O, FeSO_4_.7H_2_O, Potassium dichromate (K_2_Cr_2_O_7_), and CdSO_4_.4H_2_O) was also evaluated. The fungal dry weight and melanin concentration were detected in each experiment, as explained previously.

### DPPH radicals scavenging efficiency

The DPPH method [45] was used with slight modifications to assess the antioxidant efficacy of the melanin compared to the standard L-DOPA. This method is based primarily on evaluating the potential of melanin to scavenge free radicals. About 1 mL of the tested specimen was dissolved in methanol and combined with 1 mL of DPPH (0.002% dissolved in methanol). The mixtures were thoroughly mixed and allowed to settle for 30 min. The fusion compounds demonstrated a high level of sharpness at 517 nm UV- spectra. Ascorbic acid served as a standard. Antioxidant efficacy was assessed as the reduction in the intensity of DPPH. DPPH radical scavenging efficiency was determined as EC50 value, where 50% of the DPPH radicals were scavenged [46].

### Detecting the inhibition of mycotoxin production

To detect the effect of purified fungal melanin on the production of AFB1 and OTA by *A. flavus* and *A. carbonarius*, respectively, Erlenmeyer flasks containing 100 mL of CD liquid medium were supplemented with varying amounts of pure melanin (50, 100, 200, 300, 400, and 500 mg). Each flask was inoculated separately with a spore suspension of *A. flavus* and *A. carbonarius* (1 × 10^6^ spores/mL). The flasks were incubated for ten days at 25 °C, the culture media were centrifuged at 10,000 rpm for 30 min, and the mycelia removed. The concentrations of aflatoxin-B1 and ochratoxin-A were detected in the mycelia filtrate as follows. The AFB1 was isolated from *A. flavus* culture filtrate following the method described by *Schuller et al*. [47], and the final extracts were purified according to the methodology described by Takeda et al. [48]. The OTA was isolated from *A. carbonarius* culture filtrate by mixing it with an equal volume of methylene chloride [49], following the removal of fat with n-hexane [50, 51]. The culture filtrate was subsequently stirred for 30 min and left to stand for an additional 30 min in a separating funnel. Following the filtration through anhydrous sodium sulfate, the methylene chloride layer was subjected to vacuum evaporation until it reached complete dryness.

HPLC analysis was further performed to measure the concentrations of mycotoxins AFB1 and OTA. This analysis was performed at the Animal Health Research Institute, Dokki, Giza, Egypt, using an Agilent Series 1200 quaternary gradient pump, autosampler, FLD detector, and HPLC 2D Chemstation software (Hewlett-Packard, Les Ulis, Germany). The chromatographic-graphic separation was performed using a reversed-phase column (Extend-C18, Zorbax column, 4.6 mm i.d., 250 mm, 5 μm, Agilent Co.) [37].

### Heavy metal adsorption

The fungal melanin was tested for its potential to adsorb the HM ions using batch experiments as described by Nguyen et al. [52]. Approximately 20 mL of 0.2 mg/mL Potassium dichromate (K_2_Cr_2_O_7_ 0.2 mg/mL Cd sulfate solutions (CdSO_4_) were prepared separately in conical glass flasks (50 mL). In the tests for detecting the impact of the initial HM ion concentration, Cr and Cd concentrations were adjusted at 5 mg/L. Throughout these experiments, melanin was used at a solid-to-liquid ratio of 0.5%, except for the one specifically designed to examine the impact of a solid-to-liquid ratio. The impact of initial pH was evaluated to select the optimal pH value for HM removal. Then, the initial pH was set at 4.0, which was the optimal choice for HM removal experiments. The mixture was thoroughly mixed with a shaker at 150 rpm for two hours at 25 °C. Afterward, the supernatant was filtered with a filter membrane (Pore size 0.45 μm), and then, the HM concentration evaluated in the filtrate using an Atomic Absorption Spectrophotometer (AAS) (Unicam 969) at the Central Laboratory, Faculty of Agriculture, Zagazig University. The removal efficiency was calculated using the following equation;

$${\text{Removal efficiency }}\left( \% \right){\text{ }} = {\text{ }}\left( {{\text{C}}0 - {\text{Ct}}} \right)/{\text{C}}0{\text{ }} \times 100$$

Where C0 is the initial ion concentration and Ct is the ion concentration at time.

In addition, the residual HM (In the precipitate), chelated with melanin, was also estimated. The precipitate (containing melanin-chelated HM) was dried in an oven for an hour. The resulting powder specimens (dry precipitate) were analyzed by FTIR spectral analysis and Energy-dispersive X-ray spectroscopy (EDX). FTIR spectral analysis of the samples was performed at room temperature with disbanding whole reflection by an FTIR (Perkin Elmer). The specimens were fused and compressed with KBr, then maintained at a rate of 650–4000 cm^−1^. Output signs have been described and analyzed using spectra software [34, 53].

EDX was performed at the Regional Center for Mycology and Biotechnology (RCMB), Al-Azhar University, Cairo, Egypt, to measure the percentage of melanin-chelated HM. An X-ray detector (Ametet) with an accelerating voltage of 20 kV was used to perform EDX Spectrometer for semi-quantitative and qualitative elemental assessment (Quanta FEG250). Based on the peak emission of X-rays produced by the interaction of each element of a compound with the electron beam, the EDX technique is useful for figuring out the composition of samples. An electron or X-ray beam is directed into the sample under study in order to induce the emission of distinctive X-rays from it. Ground state (or unexcited) electrons in distinct energy levels or electron shells attached to the nucleus are present in an atom in the sample when it is at rest. An electron in an inner shell may be excited by the incident beam, which would cause it to be ejected from the shell and leave an electron hole in its place. Following the filling of the hole by an electron from an outer, higher-energy shell, the energy differential between the higher- and lower-energy shells may be emitted as an X-ray. An energy-dispersive spectrometer can detect the quantity and energy of X-rays released from a specimen. EDS enables the measurement of the specimen’s elemental composition since the X-ray energies are indicative of the energy differential between the two shells and the atomic structure of the emitting element [54].

### In-silico study

#### Molecular docking study

The study investigated molecular interactions between melanin and two fungal toxins through *in silico* approaches. The crystal structure of aflatoxin from *Aspergillus flavus* was obtained from the Data Bank (PDB ID: 8hbs), while the ochratoxin structure from *Aspergillus carbonarius* was retrieved from UniProt (ID: AF-A0A1R3RGJ2-F1-model_v4). Three-dimensional ligand structures were generated using ChemBio Office software in conjunction with the Drug Bank database. Ligands preparation involved removing water molecules and co-crystallized ligands using UCSF Chimera, followed by the addition of polar hydrogen atoms and assignment of partial charges. AutoDock Vina software was employed for molecular docking simulations to analyze binding poses and calculate interaction energies between the ligands and target toxins [55].

#### Molecular dynamics simulations

The toxin-ligand complexes from docking studies were placed in water boxes with counter ions for system neutralization. Energy minimization was performed to eliminate steric clashes before running molecular dynamics simulations in UCSF Chimera to assess complex stability over time. The analysis included evaluation of docking scores and hydrogen bonding patterns [55].

### Statistical analysis

Data are presented as mean ± SD, based on triplicate measurements from three independent experiments. Different letters (a–h): A statistically significant difference at *P* < 0.05 according to One-way ANOVA with LSD and Duncan [56]. Using, i: A statistically significant difference at *P* < 0.05 according to Independent *t*-test. *: A statistically significant difference at *P* < 0.05

## Results

### Fungal isolates and melanin productivity

As shown in Supplementary data Figure S1, all the isolates tested exhibited the ability to produce melanin. However, the *A. flavus* and *A. carbonarius* produced the highest amounts of melanin pigment about 690 µg/mL and 517 µg/mL, respectively. The most efficient melanin producer, isolate *A. flavus*, was subsequently confirmed by 18S-28S rRNA gene sequencing. It showed the highest sequence similarity with the strain A. flavus ATCC16883 (NR_111041). The phylogenetic tree was constructed (Fig. S2), and the sequences were deposited into the GenBank (https://www.ncbi.nlm.nih.gov/Genbank) under accession No. MZ314535.

### Identification of melanin characteristics

#### Physicochemical properties

The melanin pigments extracted from *A. flavus* and *A. carbonarius* were primarily identified by the physicochemical properties compared to the standard L-DOPA. The extracted melanin pigments were insoluble in hot and cold distilled water and various organic solvents, including methanol, ethanol, hexane, chloroform, benzene, acetone, and ethyl acetate (Table S1). In addition, melanin pigments dissolved rapidly in KOH at 100℃. Then, the pigments underwent condensation at pH 3 and 3N HCl, forming flocculent brown condensate in the FeCl_3_ test. When the extracted melanin was exposed to reactions with oxidizing chemicals such as H_2_O_2_, NaOCl, KMnO_4_, and K_2_Cr_2_O_7_, these agents bleached it and became colorless. In contrast, the pigments become decolorized and change to brown when interacting with the reducing agent’s potassium ferricyanide and sodium dithionite (Table S1).

#### Melanin pathways

**Fig. 1:**
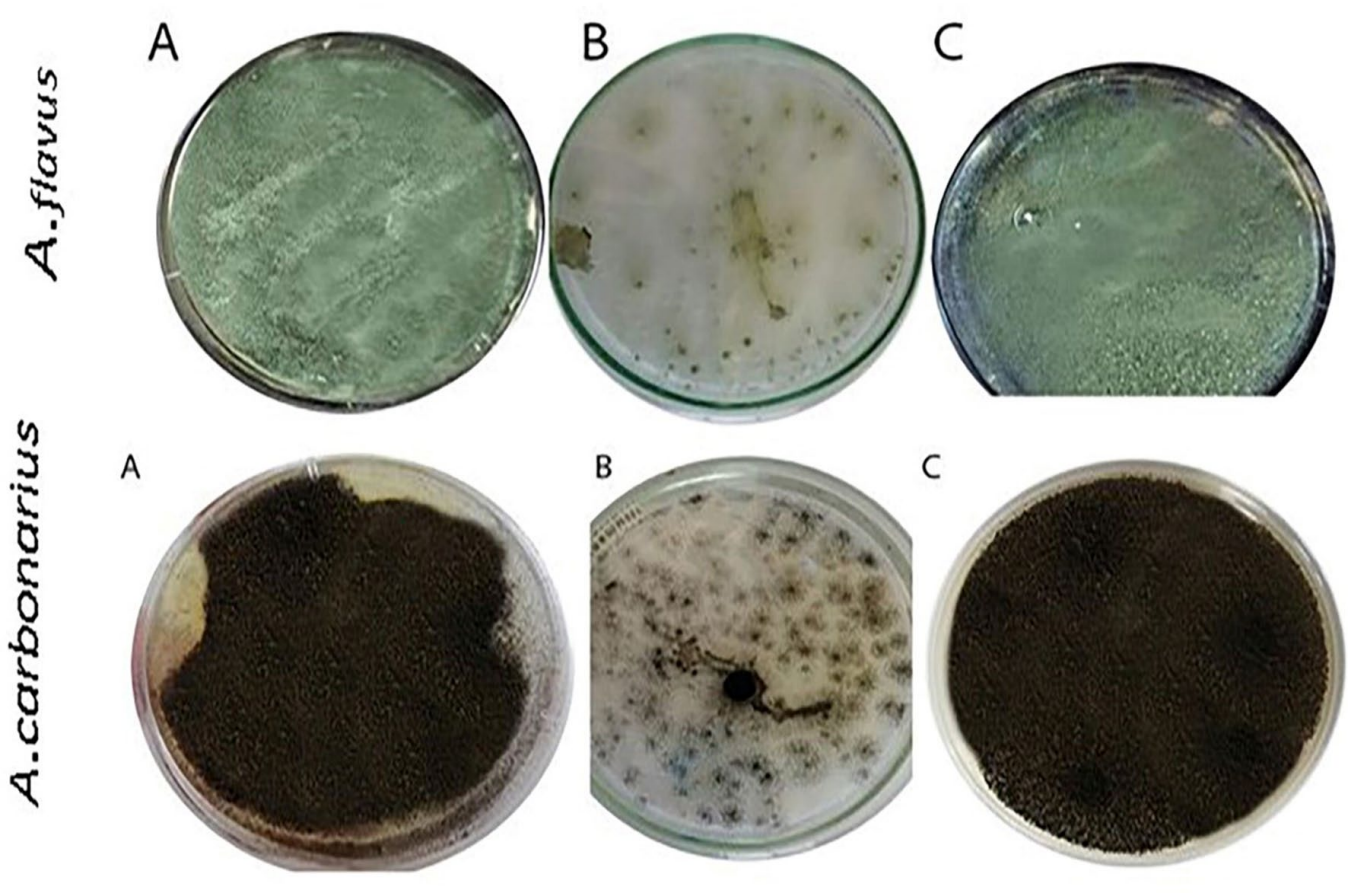
Inhibitor effect on *A. flavus* and *A. carbonarius* melanin biosynthesis pathway; **A** the fungus (control), **B** The fungus with kojic acid, **C** The fungus with tricycalazole

Melanin formation was verified through the identification of DOPA and DHN pathways in *A. flavus* and *A. carbonarius* (Fig. 1). The pathway output and efficient suppression of melanin preparation exhibited during total discoloration of the fungal strains were acquired in the consistency of an inhibitor for the tested strains. The inhibitory effects of the pathway output and effective suppression of melanin preparation were observed during the complete discoloration of the fungal strains.

#### UV, HPLC, FTIR, and ^1^H-NMR analyses

**Fig. 2:**
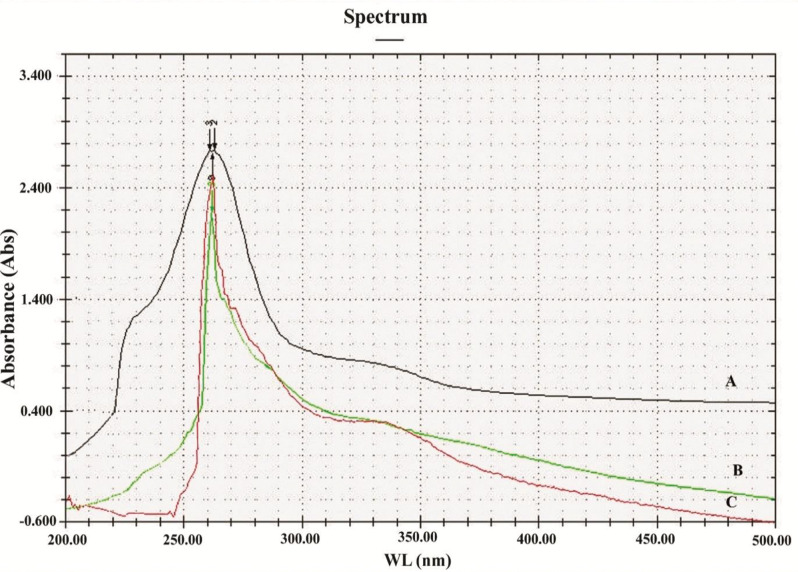
UV absorbance profiles at wave length (WL) 260 nm of standard synthetic melanin (**A**), *A. flavus* (**B**) and *A. carbonarius* melanin (**C**)

The UV-Vis analysis of the melanin produced from A. flavus showed an optical density of 3.5 at 260 nm, similar to that of the standard (L-DOPA), followed by the melanin produced by *A. carbonarius* (Fig. 2).

**Fig. 3:**
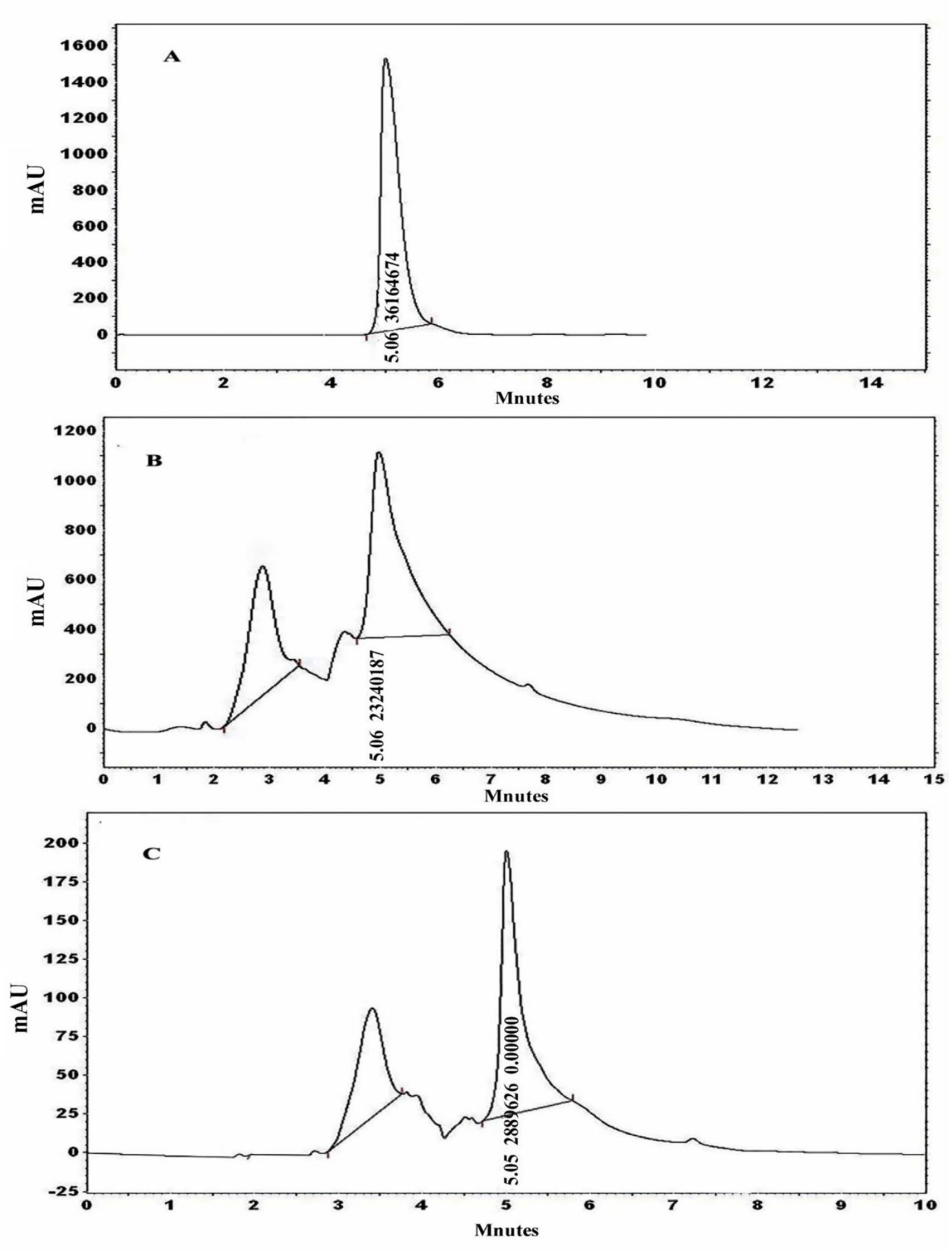
HPLC analysis for standard synthetic melanin (**A**), *A. flavus* (**B**) and *A. carbonarius* melanin (**C**)

The HPLC analysis of both the fungal melanin and the standard L-DOPA revealed a distinct peak with a retention time of approximately 5.06 min in the upper region of the curve. This peak suggests that the purity of *A. flavus* and *A. carbonarius* melanin is comparable to that of the standard melanin, as depicted in Fig. 3A, B and C.

**Fig. 4:**
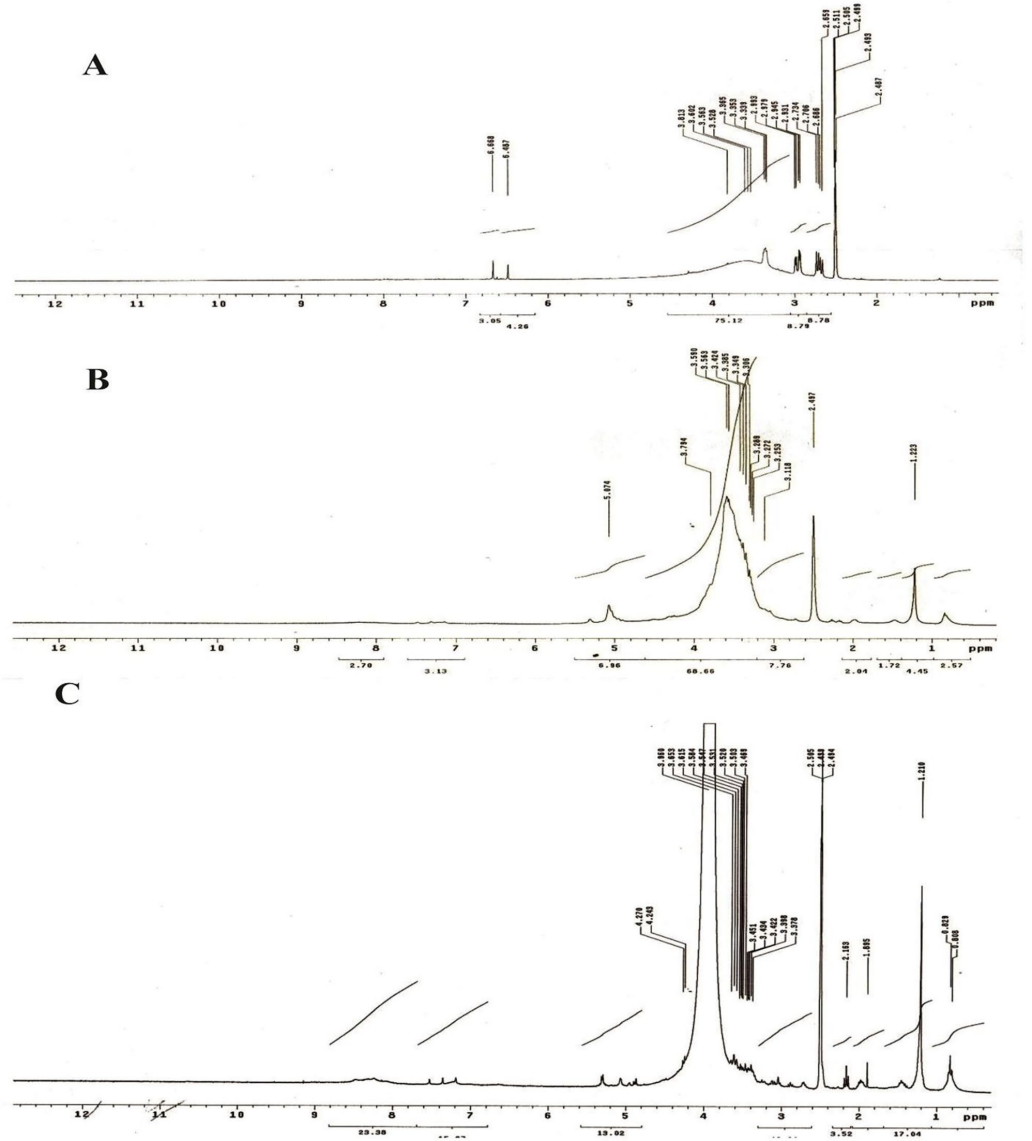
HNMR spectra of standard synthetic melanin (**A**), *A. flavus* (**B**) and *A. carbonarius* melanin (**C**)

Moreover, the 1H-NMR spectroscopy was used to identify the melanin produced by *A. flavus* and *A. carbonarius*. The analysis showed that the pigments were detected within the range of 1.0 to 8.0 ppm (Fig. 4A, B and C). The ^1^H-NMR spectrum of extracted melanin exhibits distinct signals in both the aromatic and aliphatic regions. The chart’s upper region displayed an increase in absorption levels, ranging from 3.79 to 5.07 ppm, designed to be carbons or protons correlated to oxygen and/or nitrogen atoms. In the aliphatic area of the extracted melanin’s ^1^H-NMR spectra, signals in the region 1.2 ppm can be proposed to CH_3_ groups of alkyl parts, such as CH_2_CH_3_ and CH(CH_3_)_2_. The compound CH (CH_3_)_2_ exhibits a consistent bond at a frequency of 6.9 Hz 25. The shift observed at the 8.7 ppm region clearly indicates the presence of an indole ring, which appeared in the L-Dopa region of 7.7 ppm.

**Fig. 5:**
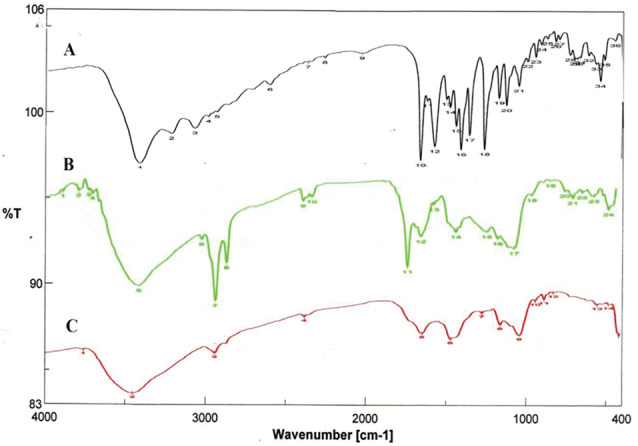
FTIR analysis for standard synthetic melanin (**A**), *A. flavus* (**B**) and *A. carbonarius* melanin (**C**)

Furthermore, the results of FTIR analysis showed that the melanin pigments of *A. flavus* and *A. carbonarius* exhibited FTIR spectra identical to those of the standard L-DOPA (Fig. 5A, B and C). The peak at around 3428 cm^−1^ on the chart is primarily attributed to the presence of (OH) and NH groups, while the peaks at 2924 cm^−1^ and 2854 cm^−1^ correspond to CH_2_ or –CH_3_ groups. Furthermore, broad signals at 1710 and 1627 cm^−1^ indicate the presence of ketone or carboxylic acid (C=O) functional groups. The bands observed at 850, 786, and 708 cm^−1^ correspond to the stretching vibrations of (C–H), =C–H, and out-of-plane bend vibrations of N-H or C–Cl. These bands indicate the presence of aliphatic compounds, while the absence of standard curve modes is indicative of the absence of aliphatic series.

#### Optimization of melanin production conditions

**Fig. 6:**
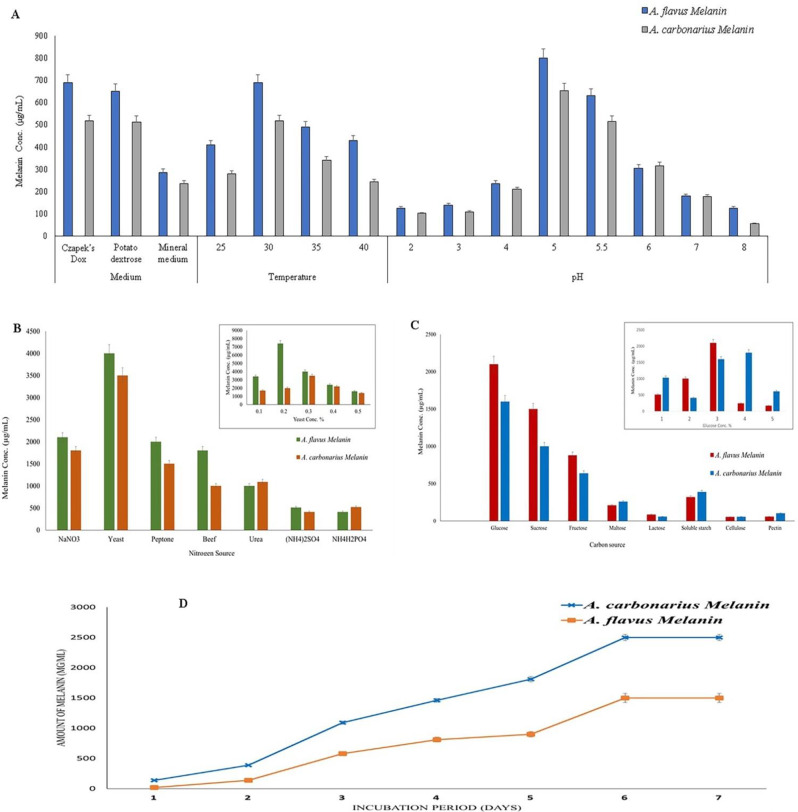
Optimization of melanin production conditions. **A** The effect of medium type, medium pH and incubation temperature, **B** Effect of different Nitrogen sources and different concentrations of yeast as optimal nitrogen source, **C** Effect of different Carbon sources and variable concentrations of glucose as optimal carbon source, and **D** The effect of diverse incubation periods on melanin production. Data are presented as mean ± SEM from three independent experiments *P* ≤ 0.05

In order to maximize melanin production by *A. flavus* and *A. carbonarius*, the production conditions, including medium, temperature, pH, carbon source, nitrogen source, and incubation period were optimized. The concentration of produced melanin was estimated in each experiment. Among the three tested media, CD was the best medium for melanin production at 30 ℃ after seven days of incubation. The amount of the produced melanin by *A. flavus* and *A. carbonarius* at 25℃ was less than the produced amount at 30℃ and upon the increase of incubation temperature over 30℃, the quantity of produced melanin decreased (Fig. 6A). The pH values of the growth medium depend on the use of citrate-phosphate buffer, and it was found that the production of melanin increased gradually with the increase of pH until reaching pH 5 where both *A. flavus* and *A. carbonarius* exhibited the maximum dry weight and melanin concentration (12,000 µg/mL and 800 µg/mL for *A. flavus* and, 9100 µg/mL and 653 µg/mL for *A. carbonarius,* respectively). Then, the dry weight and melanin concentration decreased steadily with the increase of medium pH until reaching the lowest level at pH 8.

By replacing the original nitrogen source of the medium with tested nitrogen sources, it was found that the production of melanin increased to the maximum level in presence of yeast extract as a nitrogen source. When diverse yeast extract concentrations were tested, it was found that the maximum melanin production by *A. flavus* obtained at 0.2% yeast extract. Conversely, *A. carbonarius* exhibited the highest melanin production when the medium was supplemented with 0.3% yeast extract (Fig. 6B). Furthermore, the production of melanin pigment in the presence of glucose, sucrose, fructose, maltose, lactose, soluble starch, cellulose, and pectin as a carbon source were tested separately. Glucose was detected as the best carbon source to augment the production of melanin by *A. flavus* and *A. carbonarius*. When several glucose concentrations were tested for their potential to enhance melanin production, it is noted that the melanin concentration increased with the increase of glucose concentration in the medium. However, the melanin production reached the highest level when the medium supplemented with 3% glucose, and the melanin production diminished with the increase of glucose amount in the medium (Fig. 6C).

The synthesis of melanin by *A. flavus* and *A. carbonarius* was examined at various time points during the cultivation process. The melanin concentration produced by both strains increased gradually with the extended incubation period at the optimal conditions until reaching the maximum level after 14 days (1500 µg/mL for *A. flavus* and 1000 µg/mL for *A. carbonarius*). When the incubation period extended to 16 days, the concentration of melanin remained constant (Fig. 6D).

Melanin synthesis by the selected fungal strains was also assessed in the optimized medium using various concentrations of HM sources. The UV-Vis analysis showed that *A. flavus* and *A. carbonarius* were able to produce melanin with the highest concentration when the medium supplemented individually with 0.5 mM CuSO_4_, 0.5 mM FeSO_4_, 0.1 mM K_2_Cr_2_O_7_, and 0.1 mM CdSO_4_. However, melanin production by both strains was found to decrease by increasing the heavy metal concentrations above the detected amounts (Table 1).

**Table 1:** Effect of heavy metal concentrations on melanin production by *A. flavus* and *A. carbonarius*

Heavy metal	Conc. (mM)	***A. flavus***		***A. carbonarius***		*p*-value Dry weight	***p-value Melanin conc.***
		Dry weight (µg/mL)	Melanin concentration (µg/mL)	Dry weight (µg/mL)	Melanin concentration (µg/mL)		
CuSO_4_	Control(0 mM)	24,000 ± 0.235^d^	7400 ± 0.02646^d^	18,100 ± 0.0577^e,i^	3500 ± 0.0741^d,i^	<0.001*	<0.001*
	0.1	24,800 ± 0.288^e^	8000 ± 0.05774^e^	18,440 ± 0.0577^f,i^	4000 ± 0.0711^f,i^	<0.001*	<0.001*
	0.2	25,000 ± 0.388^f^	8800 ± 0.0100^f^	18,880 ± 0.0588^g,i^	6100 ± 0.0773^g,i^	<0.001*	<0.001*
	0.5	29,000 ± 0.2881^h^	10,500 ± 0.0057^h^	19,300 ± 0.0244^h,i^	8300 ± 0.0577^h,i^	<0.001*	<0.001*
	1	27,100 ± 0.0571^g^	10,100 ± 0.0577^g^	16,000 ± 0.0288,^i^	3900 ± 0.0577^e,i^	<0.001*	<0.001*
	2	15,000 ± 0.0571^c^	7000 ± 05774^c^	11,000 ± 0.0118^c,i^	1200 ± 0.0611^c,i^	<0.001*	<0.001*
	3	10,330 ± 0.173^b^	4300 ± 0.0529^b^	7800 ± 0.0551^b,i^	600 ± 0.0244^b,i^	<0.001*	<0.001*
	5	7533 ± 0.01732^a^	1010 ± 0.1039^a^	4700 ± 0.0579^a,i^	120 ± 0.0177^a,i^	<0.001*	<0.001*
FeSO_4_	Control (0 mM)	24,000 ± 0.241^e^	7400 ± 0.0544^e^	18,100 ± 0.111^e,i^	3500 ± 0.0571^e^	<0.001*	<0.001*
	0.1	25,500 ± 0.147^f^	8000 ± 0.0571^f^	19,867 ± 0.197^f,i^	4100 ± 0.0583^f^	<0.001*	<0.001*
	0.2	26,260 ± 0.209^g^	9010 ± 0.011^g^	21,333 ± 0.0381^g,i^	5100 ± 0.055^g^	<0.001*	<0.001*
	0.5	28,230 ± 0.0571^h^	9510 ± 0.0551^h^	24,000 ± 0.384^h,i^	7200 ± 0.0588^h^	<0.001*	<0.001*
	1	16,333 ± 0.0381^d^	6300 ± 0.0571^d^	17,000 ± 0. 5771^d,i^	3000 ± 0.061^d^	<0.001*	<0.001*
	2	11,333 ± 0.0322^c^	5100 ± 0.0432^c^	13,000 ± 0.5772^c,i^	2100 ± 0.0561^c^	<0.001*	<0.001*
	3	7767 ± 0.0681^b^	3800 ± 0.0477^b^	8167 ± 0.579^b,i^	1560 ± 0.064^b^	<0.001*	<0.001*
	5	3100 ± 0.0571^a^	1200 ± 0.0117^a^	3167 ± 0.0172^a,i^	800 ± 0.0633^a^	0.406	<0.001*
K_2_Cr_2_O_7_	Control (0 mM)	24,133 ± 0.1271^d^	7400 ± 0.5773^e^	18,133 ± 100.00^e,i^	3510 ± 0.001^d,i^	<0.001*	<0.001*
	0.1	53,000 ± 0.5410^h^	16,300 ± 0.611^h^	40,333 ± 0.100^h,i^	9700 ± 0.057^h,i^	<0.001*	<0.001*
	0.2	48,667 ± 0.2410^g^	12,100 ± 0.711^g^	39,267 ± 0.0572^g,i^	8100 ± 0.056^g,i^	<0.001*	<0.001*
	0.5	40,667 ± 0.0577^f^	10,000 ± 0.0521^f^	37,333 ± 0.0571^f,i^	6200 ± 0.057^f,i^	<0.001*	<0.001*
	1	29,333 ± 0.3821^e^	6200 ± 0.0577^d^	17,667 ± 0.0573^d,i^	4100 ± 0.573^e,i^	0.321	<0.001*
	2	22,333 ± 0.3841^c^	4810 ± 0.0573^c^	13,267 ± 0.0561^c,i^	1900 ± 0.0573^c^	<0.001*	<0.001*
	3	18,600 ± 0.5711^b^	1210 ± 0.5743^b^	10,667 ± 0.0531^b,i^	1120 ± 0.5773^b,i^	<0.001*	<0.001*
	5	8900 ± 0.5712^a^	880 ± 0.0571 ^a^	8133 ± 0.0051^a,i^	930 ± 0.0441^a,i^	<0.001*	<0.001*
CdSO_4_	Control (0 mM)	23,800 ± 0.0171^h^	7410 ± 0.0674^h^	18,100 ± 0.0574^h,i^	3500 ± 0.1774^h,i^	<0.001*	<0.001*
	0.1	21,000 ± 0.0722^g^	3900 ± 0.0574^g^	13,000 ± 0.0774^g,i^	3000 ± 0.0774^g,i^	<0.001*	<0.001*
	0.2	18,330 ± 0.0572^f^	3330 ± 0.0774^f^	8100 ± 0.0574^f,i^	2610 ± 0.5773^f,i^	<0.001*	<0.001*
	0.5	14,470 ± 0.0103^e^	2130 ± 0.0273^e^	6233 ± 0.0574^e,i^	1810 ± 0.5773^e,i^	<0.001*	<0.001*
	1	13,267 ± 0.011^d^	1830 ± 0.0774^d^	5167 ± 0.0474^d,i^	1260 ± 0.2774^d,i^	<0.001*	<0.001*
	2	10,060 ± 0.0571^c^	1560 ± 0.0874^c^	1200 ± 0.0504^c,i^	1130 ± 0.0373^c,i^	<0.001*	<0.001*
	3	8100 ± 0.0355^b^	1130 ± 0.0874^b^	800 ± 0.03773^b,i^	830 ± 0.05771^b,i^	<0.001*	0.003*
	5	5233 ± 0.07735^a^	130 ± 0.0577^a^	737 ± 0.05541^a,i^	100 ± 0.0577^a,i^	<0.001*	<0.001*

Mean in the same row having different letters are significantly different (*P* ≤ 0.05)

i: A statistically significant difference at *P* ≤ 0.05 according to Independent t-test

*A statistically significant difference at *P* ≤ 0.05

### Antioxidant activity

The antioxidant activity of fungal melanin was confirmed using the DPPH method. The purified melanin pigments exhibited significant capability to scavenge free radicals, as demonstrated by their DPPH (EC50 of 55.5 µg/mL) efficacy, which was comparable to that of L-DOPA (EC50 of 59.5 µg/mL), particularly at a concentration of 100 µg/mL. In Table 2, L-DOPA and ascorbic acid were used at concentrations of 20, 40, 60, 80, 100, 120, and 140 µg/mL. The ascorbic acid displayed a remarkable ability to scavenge radicals, with an EC50 value of 40.6 µg/mL. The DPPH scavenging activity increased with the increase in melanin concentration. According to the data in Table 2, the EC50 activity rises with the concentration increase until it reaches into stabilizing point at 100 µg/mL.

**Table 2:** Antioxidant DPPH activity of melanin produced by *A. flavus* and *A. carbonarius*

Melanin Conc. (µg/mL)	DPPH radical scavenging %				***P1*** ***Ascorbic acid***	***P2*** ***L-DOPA***	***P3 A. flavus melanin***	***P4*** ***A. carbonarius melanin***
	Ascorbic acid (Positive control)	L-DOPA (standard)	*A. flavus* melanin	*A. carbonarius* melanin				
0	0.0000 ± 0.0000^a^	0.0000 ± 0.0000^a^	0.0000 ± 0.0000^a^	0.0000 ± 0.0000^a^	-	-	-	-
20	17.3433 ± 0.05774^b^	41.07 ± 0.00577^bi^	16.57 ± 0.05774^bi^	31.12 ± 0.00577^bi^	<0.001*	<0.001*	<0.001*	<0.001*
40	24.5000 ± 0.05774^c^	46.44 ± 0.0577^ci^	31.87 ± 0.05774^ci^	34.46 ± 0.02887^ci^	<0.001*	<0.001*	<0.001*	<0.001*
60	29.1667 ± 0.05774^d^	53.63 ± 0.0577^di^	33.07 ± 0.05774^di^	41.89 ± 0.00577^di^	<0.001*	<0.001*	<0.001*	<0.001*
80	36.9167 ± 0.0577^e^	55.87 ± 0.0577^e^	48.76 ± 0.00577^ei^	47.87 ± 0.00577^ei^	<0.001*	<0.001*	<0.001*	<0.001*
100	40.6133 ± 0.0577^h^	59.59 ± 0.04041^hi^	55.51 ± 0.00577^hi^	50.78 ± 0.00577^hi^	<0.001*	<0.001*	<0.001*	<0.001*
120	40.3667 ± 0.05774^g^	58.30 ± 0.05771^gi^	54.73 ± 0.05774^gi^	50.73 ± 0.00577^gi^	<0.001*	<0.001*	<0.001*	<0.001*
140	40.7200 ± 0.0577^f^	57.800 ± 0.05771^fi^	54.71 ± 0.00577 ^gi^	50.71 ± 0.0577^fi^	<0.001*	<0.001*	<0.001*	<0.001*
160	40.7100 ± 0. 0577^f^	57.91 ± 0.00577^fi^	54.13 ± 0.05774^fi^	50.71 ± 0.05774^fi^	<0.001*	<0.001*	<0.001*	<0.001*

*Mean in the same row having different letters are significantly different (*P* ≤ 0.05)

### Suppression of mycotoxin production

**Fig. 7:**
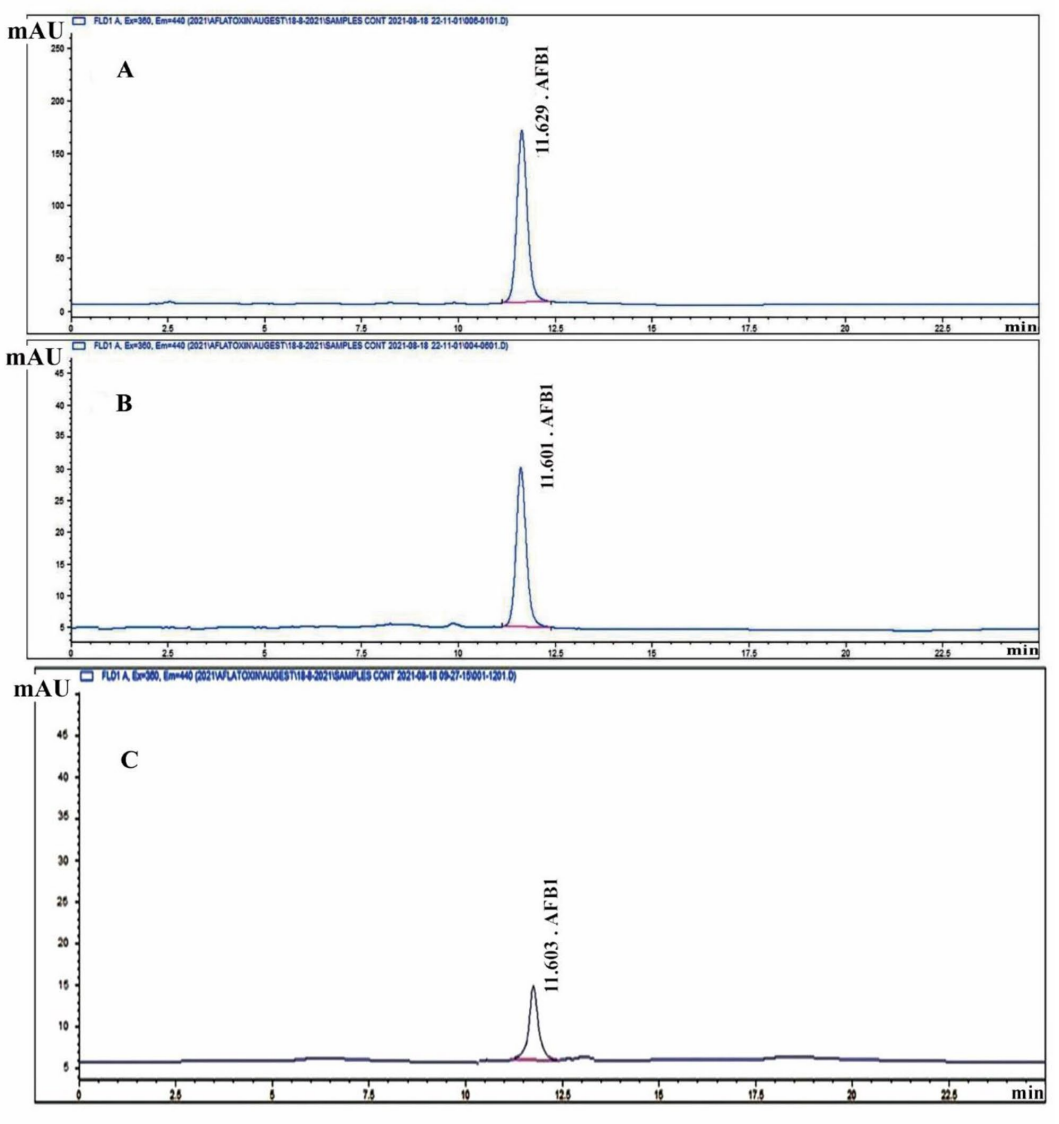
HPLC of Aflatoxin; **A** HPLC of standard of Aflatoxin B1 (40 ppb); **B** HPLC for control for *A. flavus* (without any melanin) (18.2 PPb) at retention time (11.6) which was the same of standard **C** HPLC for sample (with melanin) (0.43 ppb) at retention time (11.6) which was the same of standard

The effect of fungal melanin on the production of mycotoxins was evaluated by HPLC analysis of mycotoxins in each treatment. Our results expressed in Table 3 reflect that raising the concentration of pure melanin gradually decreases the concentration of produced Af–B1 toxin and the growth (expressed as dry weight) of *A. flavus*. It was observed that supplementing *A. flavus* culture with 0.3% pure melanin resulted in complete inhibition of AF–B1 toxin production with significant diminution in *A. flavus* growth. In addition, the results demonstrate that the increase of melanin concentration above 0.3% leads to continual decline of fungal growth. These findings were monitored by HPLC analysis of the produced AF–B1 toxin in each treatment compared to the standard toxin sample at the same retention time (5.55 min) (Fig. 7A, B and C).

**Table 3:** Effect of melanin on growth and Aflatoxin B1and Ochratoxin A production

Concentration of melanin (mg)	***A. flavus***		***A. carbonarius***		*P*-value Dry weight	*p*-value Melanin concentration
	Dry weight (mg mL^−1^)	Aflatoxin production (μg mL^−1^)	Dry weight (mg mL^−1^)	Ochratoxin production (μg mL^−1^)		
0	0.4533 ± 0.00577^g^	10.2433 ± 0.00574^e^	0.45 ± 0.00577^c^	87.34 ± 0.00577^fh^	0.130	<0.001*
Control + solvent	0.4533 ± 0.00577^g^	10.2367 ± 0.06351^e^	0.45 ± 0.00577^c^	87.3 ± 0.0083^fh^	0.159	<0.001*
0.05	0.3900 ± 0.01000^f^	8.2467 ± 0.00577^d^	0.3433 ± 0.00577^bch^	23.8800 ± 0.06928^eh^	0.001*	<0.001*
0.1	0.3367 ± 0.00577^e^	3.6600 ± 0.01000^c^	0.2633 ± 0.00577^abh^	8.6233 ± 0.00577^dh^	<0.001*	<0.001*
0.2	0.2700 ± 0.01000^d^	1.3567 ± 0.04619 ^b^	0.3900 ± 0.00511^bch^	7.0533 ± 0.00577^ch^	<0.001*	<0.001*
0.3	0.1533 ± 0.00155^c^	0.0000 ± 0.00000^a^	0.1633 ± 0.01528^ab^	1.9467 ± 0.01528^bh^	0.036	<0.001*
0.4	0.0000 ± 0.00000^a^	0.0000 ± 0.00000^a^	0.0867 ± 0.01155^ah^	0.0000 ± 0.00000^ah^	<0.001*	-
0.5	0.0600 ± 0.00100^a^	0.0000 ± 0.00000^a^	0.0500 ± 0.01000^ah^	0.0000 ± 0.00000^ah^	<0.001*	-

*Mean in the same row having different letters are significantly different (*P* ≤ 0.05)

**Fig. 8:**
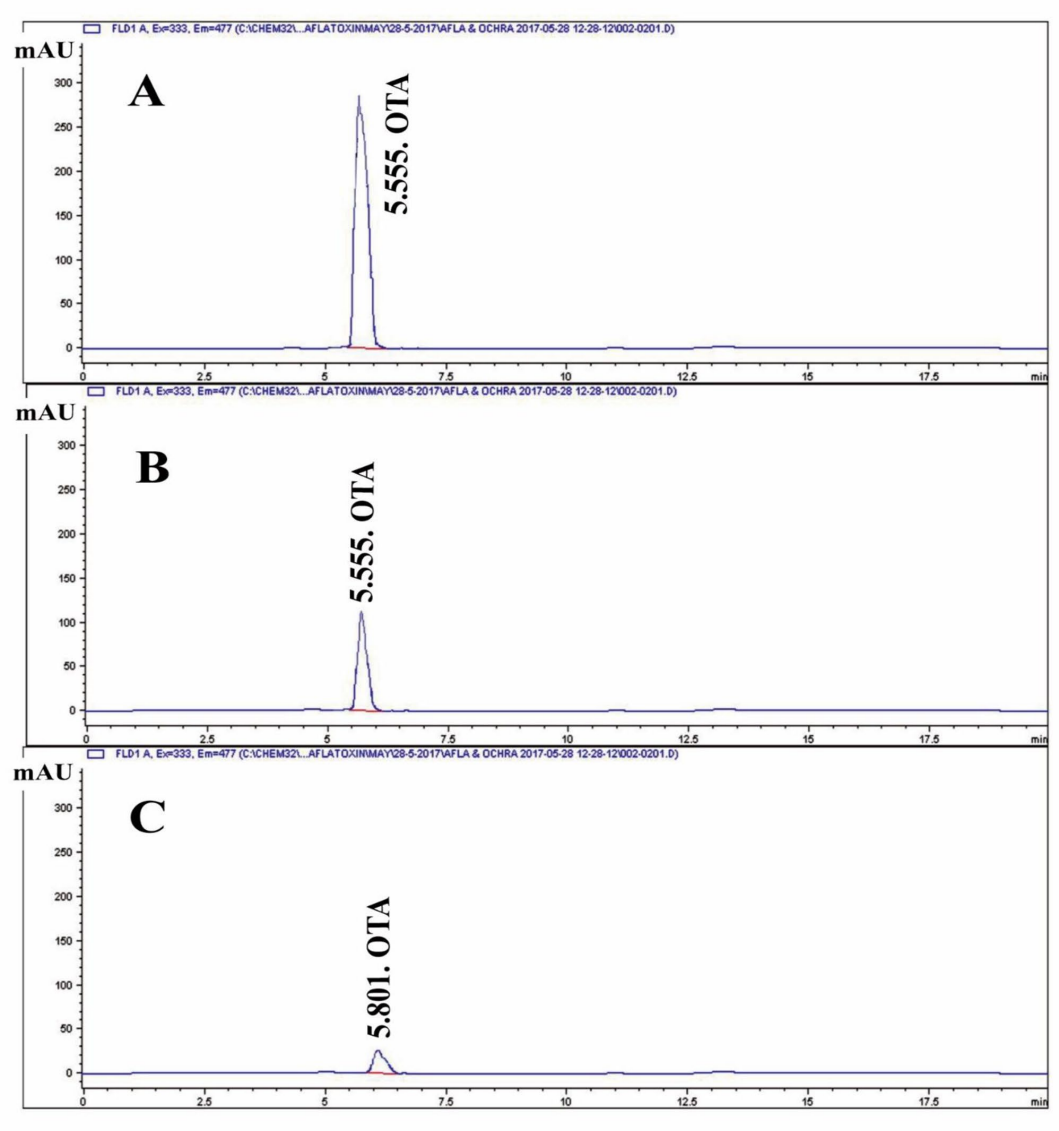
HPLC of Ochratoxin; **A** HPLC for standard of Ochratoxin; **B** HPLC for control for *A. carbonarius* (without any melanin) (15.84 PPb) at retention time (5.55) which was the same of standard **C** HPLC for sample for *A. carbonarius* (with pure melanin) (3.04 PPb) at retention time (5.55) which was the same of standard

The effect of melanin on the production of OTA by *A. carbonarius* is illustrated in (Table 3). Evidently, the increase of melanin in the culture medium results in the continuous decline in OTA and the decline of fungal growth. By measuring the produced OTA in each experiment, we found that 0.4% melanin-enriched medium caused complete suppression of OTA production, and the increased melanin concentration in the medium of *A. carbonarius* continued to reduce the fungal growth. Our findings were confirmed by HPLC analysis of OTA in each experiment output. The OTA produced in each treatment was analyzed in comparison with the standard OTA at the same retention time (5.55 min) (Fig. 8A, B and C).

### Heavy metal chelation

The efficiency of purified fungal melanin to chelate the heavy metals Cd and Cr was identified using AAS analysis for the experiment solution filtrate and EDX and FTIR analyses for the melanin-chelated HM powder. The results demonstrated a direct relationship between the concentration of melanin and the adsorption of Cr and Cd. The potential of melanin to adsorb heavy metals was calculated and expressed as a removal efficiency percentage. It was found that the addition of 1 mg/mL of purified melanin resulted in a 49% removal efficiency of Cd and 63% of Cr. Our results showed that the removal efficiency of Cd increases up to 56%, 57%, and 60% with the increase of melanin concentration to 5, 10, and 15 mg/mL, respectively. The presence of comparable melanin concentrations resulted in the removal efficiency of Cr to 64%, 67%, and 77 %, respectively (Table 4).

**Table 4:** Cadmium and chromium adsorption efficiency by melanin

Melanin Conc. (mg)	Metal removal efficiency (%)		*p*-value
	Cd	Cr	
1	49.4333 ± 0.00547^d^	63.70 ± 0.0001^d,e^	<0.001*
5	56.1000 ± 0.00321^c^	64.63 ± .57735^c,e^	<0.001*
10	57.1667 ± 0.00275^b^	67.33 ± 0.05774^b,e^	<0.001*
15	60.0333 ± 0.01774^a^	77.13 ± 0.05774^a,e^	<0.001*

Mean in the same row having different letters are significantly different (*P* ≤ 0.05)

**Fig. 9:**
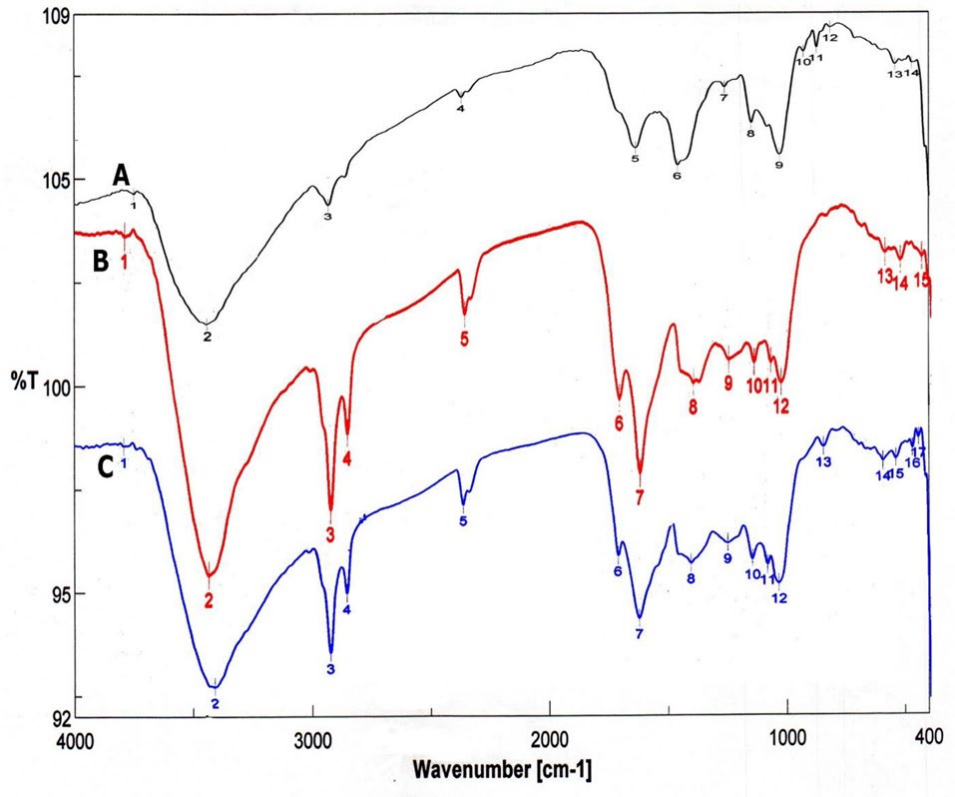
FTIR (Peak shift) for adsorption efficiency of melanin **A** control of pure melanin, **B** 15 mg of melanin with Cd, **C**15 mg of melanin with Cr

The FTIR analysis of melanin-chelated Cd, compared to the control sample of pure melanin, showed a shift in the occurrence of specific peaks and the appearance and disappearance of new peaks (Table S2, Fig. 9A and B). The presence of broken bonds at peaks 3736 and 3434 cm^−1^ indicates the strong presence of Alcohol (O-H) bands. Also, a new peak at 2853 cm^−1^ was observed, representing the C–H bonds in the alkane group, which was absent in the control specimen (Fig. 9B). Another new peak at 1712 cm^−1^ implies the stretching mode of C=O bonds, which could be attributed to either ketone or carboxylic acid. The intense peaks at 2853, 1710, 596, and 447 cm^−1^ correspond to alkanes, the C=O stretching mode of a ketone or carboxylic acid, and the C–Br and C–I stretching, respectively. A shift of 50 cm^−1^ at 1456.03 cm^−1^ signifies a change in the C–H bending in CH_3_ or C=C groups (scissoring) or aromatic –C=C stretching vibrations. The shift at 3408 cm^−1^ (Δ 26 cm^−1^) corresponds to the stretching of the –OH alcohol group (O–H), which is firmly and broadly H-bonded. The shift at 1632 cm^−1^ (Δ 10 cm^−1^) can be attributed to the stretching mode of C=C, N-H bending in primary amine, or C=O stretching mode (amide). The consecutive shifts at 2361, 1623, and 1260 cm^−1^ (Δ 2, 10, 8 cm^−1^) back to the stretching mode of C¼O in carbonyl groups found in alcohol, esters, ethers, carboxylic acids, as well as the stretching mode of C≡N and N–H bending in primary amine. The intensity of the peak diminished, and a slight displacement occurred at 1147 cm^−1^, corresponding to the C-O stretching mode (ether) or C–O stretching mode. Furthermore, the peaks observed at 928 cm^−1^ and 815 cm^−1^ were no longer present in the sample (26-b).

The FTIR spectral analysis of melanin-chelated Cr, compared to the pure fungal melanin, represented in Fig. 9A and C, revealed a significant shift in the woven Umber at 3785 and 3408 cm^−1^ (Δ49 and 26 cm^−1^, respectively). This shift indicates the interaction between –OH groups and the absorption of Cd, as well as the asymmetric stretching of –NH_2_ groups in amines. The prominent peaks observed at 2853 cm^−1^ and 1710 cm^−1^ (Fig. 9C) corresponds to the stretching vibrations of the C–H bonds in the alkane group (strong, intense) and the stretching vibrations of the O–H bonds in the carboxylic group. These peaks also show the stretching vibrations of the C=O bonds in ketones and carboxylic acids. A further significant change was observed at 1456 cm^−1^ (Δ 50) cm^−1^ as a result of aromatic –C=C stretching vibrations and C–H bending in CH3 groups (scissoring). The reasons for the significant shift at 1260 cm-1 (Δ 8 cm^−1^) were C=O bending mode, C–O–H bending mode, C–O stretching mode (ether), C–O stretching mode (alcohol), and C–O carboxylic acid. At 873 cm^−1^ (Δ 24) cm^−1^, another noticeable shift was identified as an indication of C–S stretching or C–Br, C–I (alkyl halides). In addition, there was a change in the peak intensity and a drop in the peak at 1147 cm^−1^, 541 cm^−1^, and 471 cm^−1^ (C–O stretching mode (ether) or C–O stretching mode and C–S stretching or C–Br, C–I (alkyl halides). The peak at 928 cm^−1^ in control disappeared. In sample c, a new C–S stretching peak resurfaced in a substantial shift band at 447 cm^−1^ (Table S3).

**Fig. 10:**
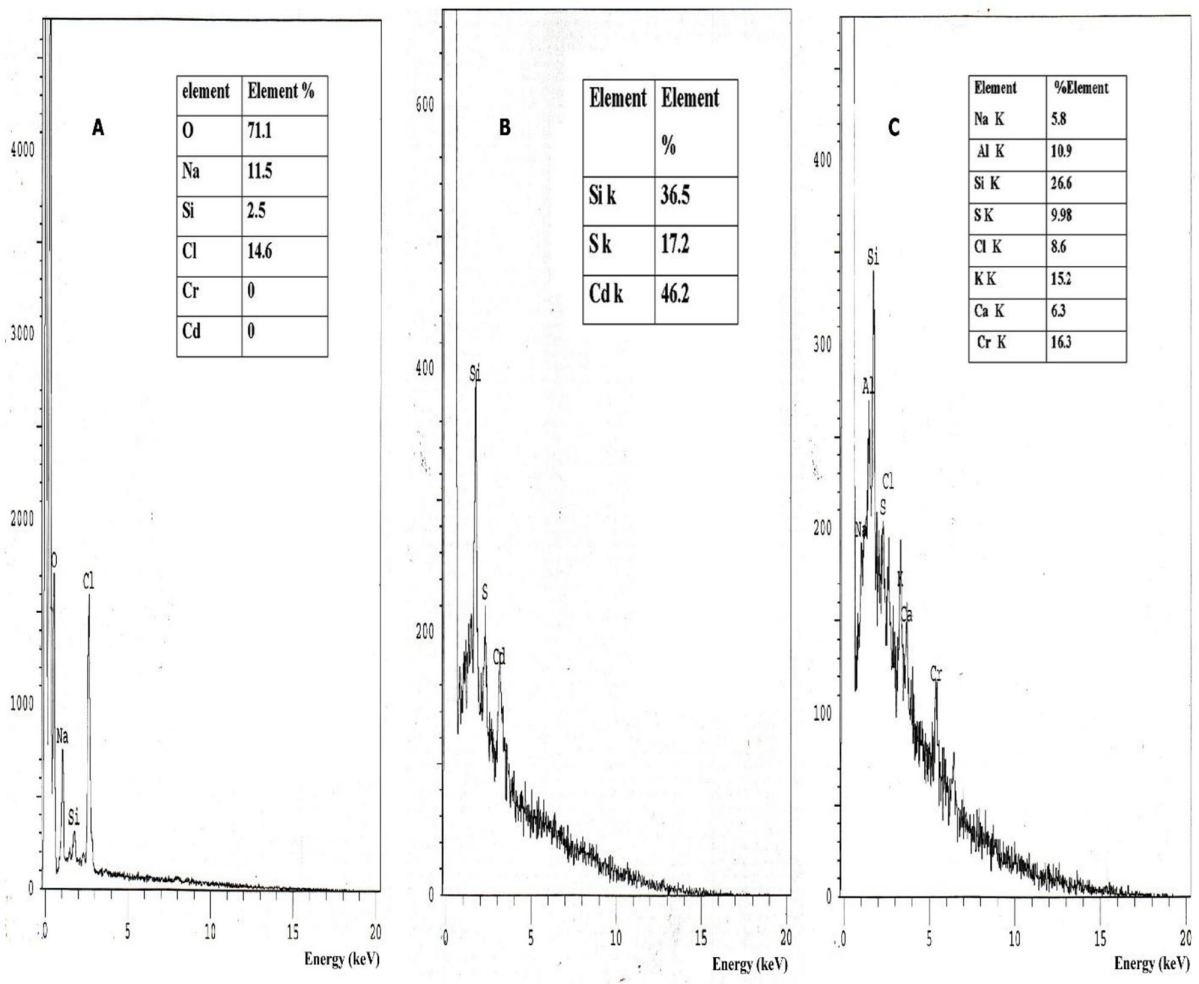
EDEX of **A** Pure melanin (control), **B** Melanin after adsorption of Cd metal ions, and **C** Melanin after adsorption of Cr metal ions

The EDX spectral analysis demonstrated the presence percentage of Cd and Cr on the melanin surface with percentages of 46.2% and 16.3%, correspondingly compared to the control sample of pure fungal melanin Fig. 10A, B and C.

The molecular docking studies revealed distinct binding characteristics between melanin and the two fungal aflatoxin and ochratoxin (Table 5).

**Table 5:** Docking scores and hydrogen bonding melanin and *aflatoxin* and *ochratoxin*

Free binding energy of the ligands, temperature (T) = 298.15 K		
	***aflatoxin in Aspergillius flavus***	***ochratoxin in Aspergillius carbonarieus***
Melanin	**−9.5 kcal/mol**	**−5.4 kcal/mol**
	Hydrogen-Oxygen Bond, 2.20 Å, from Tyr 180 Hydrogen to Ligand Oxygen	No Hydrogen Bonds

**Fig. 11:**
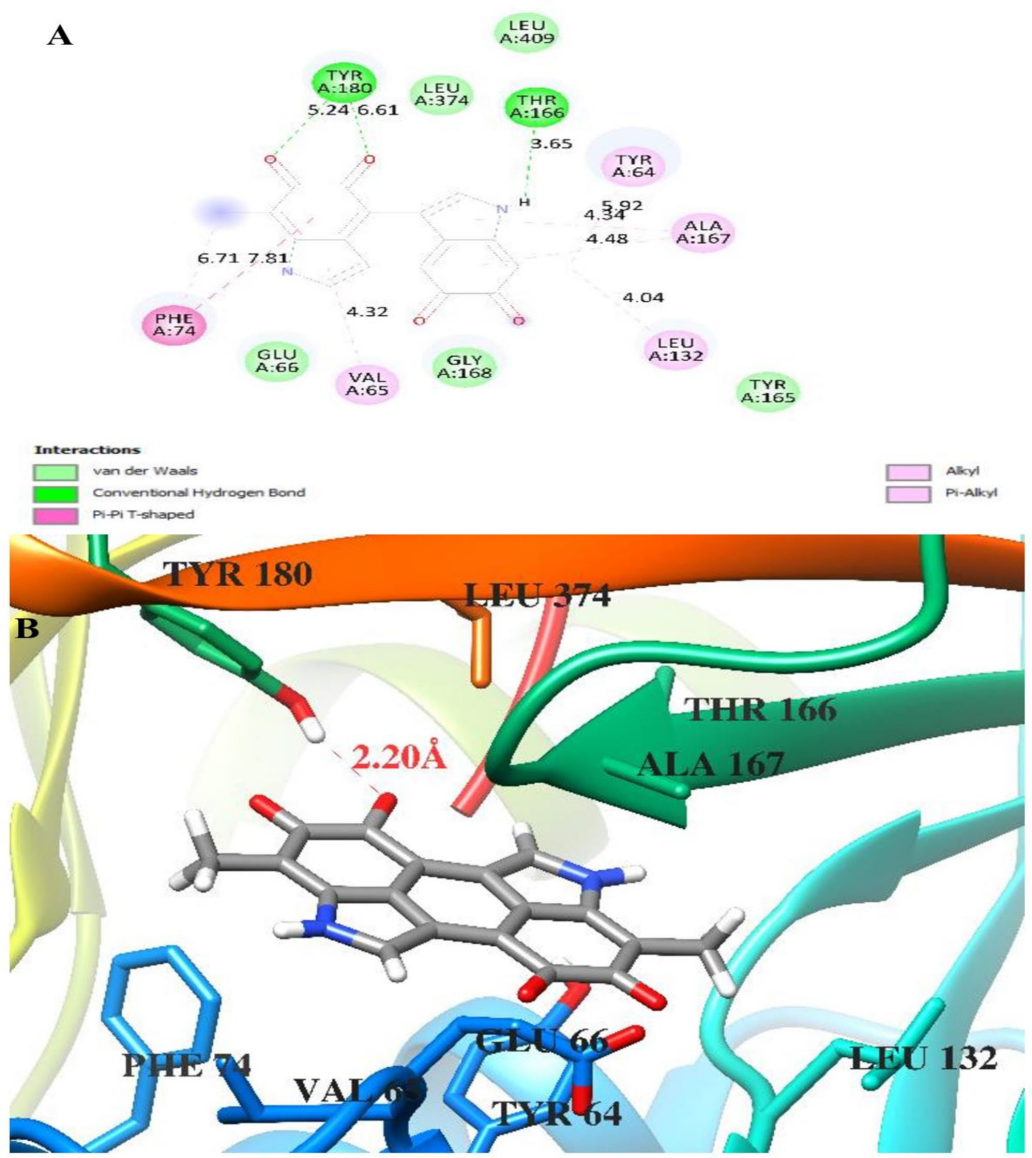
Docking of Melanin on *aflatoxin in Aspergillius flavus*** A** Aflatoxin backbone in ribbon representation with key amino acid residues and **B** Molecular docking visualization and interaction diagram of melanin binding to aflatoxin in *A. flavus*

The interaction between melanin and aflatoxin in *A. flavus* showed particularly strong binding, with a free binding energy of −9.5 kcal/mol. This complex was stabilized by a specific hydrogen-oxygen bond measuring 2.20 Å between the hydrogen of Tyrosine 180 and the ligand’s oxygen atom, as clearly visible in the molecular visualization (Fig. 11A). This strong binding energy and the presence of a specific hydrogen bond suggest a stable and potentially biologically significant interaction. Where, the aflatoxin backbone is shown in ribbon representation with key amino acid residues displayed in stick format. The interaction diagram details the network of molecular contacts in the binding site, with different types of interactions color-coded according to their nature (Fig. 11A). Also, the results showed the molecular docking visualization of melanin binding to aflatoxin in *A. flavus* and the binding pocket with key structural features including the hydrogen-oxygen bond (2.20 Å) between Tyrosine 180 (TYR 180) and the melanin ligand (Fig. 11B).

**Fig. 12:**
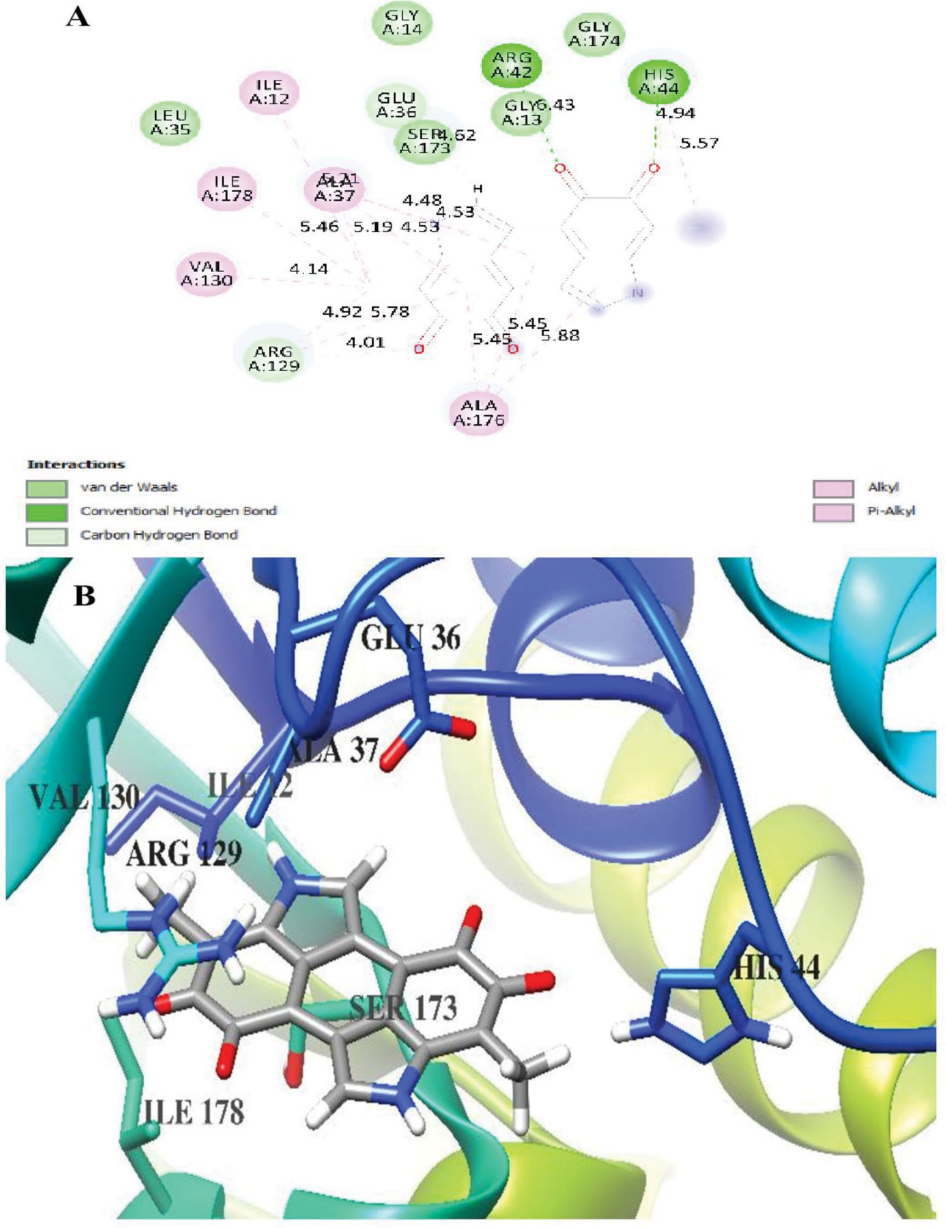
Docking of Melanin on ochratoxin in *Aspergillius carbonarieus*** A** Ochratoxin backbone in ribbon representation with key amino acid residues and **B** molecular docking visualization and interaction diagram of melanin binding to ochratoxin in *Aspergillius carbonarieus*

In contrast, the interaction between melanin and ochratoxin in *A. carbonarius* was notably weaker, with a binding energy of −5.4 kcal/mol. Interestingly, no hydrogen bonds were observed in this complex, as shown in the corresponding molecular visualization (Fig. 12A). The binding site analysis revealed the presence of several key amino acid residues including VAL 130, ARG 129, GLU 36, and HIS 44, which may contribute to the overall binding stability through other types of molecular interactions despite the absence of hydrogen bonds. The ochratoxin is represented in ribbon format with varying colors indicating different secondary structure elements, while key residues are shown in stick representation. The accompanying interaction diagram maps the spatial arrangement of protein-ligand contacts in the binding site, though no hydrogen bonds are present in this complex (Fig. 12A). Further, the results showed the molecular docking visualization of melanin binding to ochratoxin in *A. carbonarius* as well as the binding pocket environment with labeled amino acid residues including VAL 130, ARG 129, GLU 36, HIS 44, ILE 178, and SER 173 (Fig. 12B).

## Discussion

The toxicity of heavy metals and fungal contamination poses hazards to human health and the environment [1–3]. When considering a solution for this growing environmental issue, melanin, a bio-polymeric pigment, can effectively scavenge free radicals, bind metal ions, and contribute to preventing the release of mycotoxin, Melanin is a bio-polymeric pigment and can act as a scavenger of free radicals, efficiently chelate metal ions, and play a role in inhibiting the secretion of mycotoxins [57–59]. Therefore, this study is designated to employ fungi as a natural source to produce melanin. In this framework, the study began with the isolation of fungi from soil samples. All isolates were found to be melanin producers in the CDA medium. However, *A. flavus* and *A. carbonarius* were the most potent producers. Similar species are previously reported as melanin producers [33, 37, 44]. The notable benefits of these two isolates lie in their ability to generate melanin in high concentrations, where *A. flavus* produced 690 µg/mL of melanin from a fungal biomass dry weight of 9233 µg/mL. *A. carbonarius* produced 517 µg/mL of melanin from mycelial dry weight of 8770 µg/mL.

Afterward, the purified melanin from *A. flavus* and *A. carbonarius* showed typical melanin characteristics. A thorough examination was conducted on the UV-Vis spectra at a wavelength of 260 nm, which is identical to the standard (L-DOPA). This result is consistent with previous findings on the absorbance rates of various fungal melanin at altitudes ranging from 200−300 nm [60, 61]. Furthermore, a log of optical density confirmed a linear bend together with negative descents [60, 62]. Fungal melanin was water and organic solvents insoluble. Nevertheless, it exhibited high solubility in alkaline solutions and precipitated in acidic solutions, forming a flocculent brown condensate with FeCl_3_. These properties were investigated in artificial and natural melanin, which have been mentioned previously [60, 63]. The detected properties of melanin in this study are attributed to the unparalleled constructions authorizing them as proton granters or recipients [33, 64]. In addition, the refined melanin manifested positive trials towards whole chemical characters [22, 35].

To further validate our results, the process of synthesizing the DOPA or DHN-melanin pathway was demonstrated by employing various inhibitor compounds. The presence of kojic acid resulted in the lack of pigmentation in *A. flavus* and *A. carbonarius*. Kojic acid inhibits the activity of the tyrosinase enzyme that promotes the conversion of tyrosine to dopaquinone through the DOPA pathway. Hence, it is involved in the production of melanin pigment, as reported in previous studies [60, 65]. They also confirmed the presence of DHN passage in both *A. terreus* and *A. tubingensis*. Moreover, diethyldithiocarbamate, tricyclazole, pyroquilon, and thalide inhibit the synthesis of DHN melanin but do not affect the production of DOPA melanin [60, 66]. In addition to the confirmatory assays of fungal melanin, HPLC analysis was conducted to assess melanin production by *A. flavus* and *A. carbonarious*, focusing on physical and chemical data. The chemical uniformity of repetitive *A. flavus* melanin was identical to that reported in [37, 67]. The chemical composition of the extracted melanin from tested fungal strains was also verified using ^1^H-NMR and FTIR spectroscopic analyses. The melanin of *A. flavus* and *A. carbonarious* was found to be identical to the standard synthetic melanin and was confirmed to be the same as the melanin reported previously by *Kumar et al*. [32]. The ^1^H-NMR spectrum of the isolated melanin revealed signals that spanned both the aliphatic and aromatic regions. The emphasis has been placed on the methyl series of alkyl groups, where signals can be attributed to carbon or proton atoms connected to nitrogen and/or oxygen atoms. Peaks can also be assigned to protons attached to substituted aromatic or heteroaromatic moieties, yielding similar results as reported previously [37, 57, 68]. The FT–IR analysis of fungal melanin explored a broad range of intensity levels and assessed the consistency of hydrogen bonding in the OH group and the expansion of the aromatic C=C structure. Moreover, peaks corresponding to the OH and NH bonds, as well as those attributed to the presence of carbonyl bonds were identified. The distinctive characteristics of the infrared spectra for melanin were the same as those stated in previous literatures [62, 69].

In different media, the measurements of melanin concentrations showed that CD culture induced the potency of the suspicious melanin by both fungal strains. Earlier studies also demonstrated that the CD culture significantly contributed to enhancing melanin production [17, 70]. Melanin production by *A. flavus* and *A. carbonarius* exhibited a gradual increase between 14–15 days of incubation at 30 ℃ and pH 5, similar to the findings of Raman et al. [61] who proved the increase in melanin production from *Aspergillus fumigatus* by about two fold after optimization and after a period of 5 days. On the other hand, Saleh et al. [71] demonstrated a maximum melanin yield from yeast strain at about 48.5 mg/L with pH 6.0 and at 22^o^C. Additionally, the majority of melanin pigment was synthesized after 10 days of incubation and was entirely produced by the end of the lag stage of fungal germination [44]. Furthermore, a high yield of melanin was achieved at a concentration of 0.1 mM FeSO_4_ [44, 61, 72]. These results confirmed that Fe increased melanin production as Fe is a substantial cofactor for manufacturing melanin by the L-DOPA pathway. Alternatively, a previous study conducted by *El-Batal and Al Tamie* [33], showed that copper Cu enhances melanin synthesis due to its significant role as a catalyst for apo tyrosinase, thereby potentially improving protein and enzyme efficacy.

After determining the optimal melanin production conditions, the antioxidant activity of melanin was assessed. The results showed that the increase in melanin concentration leads to a gradual increase in DPPH scavenging activity by capturing electrons and scavenging ROS while enhancing the release of catalyst minerals [68]. The obtained data revealed that the melanin extracted from *A. flavus* has a high scavenging activity at 100 μg/mL (94 %). The melanin’s high concentrations effectively counteracted the presence of free radicals, which fits with the previous findings [73, 74]. The ability of melanin to remove reactive oxygen species (ROS) has been highlighted, suggesting that melanin may protect pigment cells from oxidative stress caused by ROS [75].

In addition, the potential of melanin to inhibit mycotoxin production was evaluated. The results show that increased melanin concentrations resulted in a continuous decrease of aflatoxin B1 and ochratoxin A and significant *A. flavus* and *A. carbonarius* growth control. These results fall in accordance with the previous studies [16, 76]. This inhibitory action of melanin is attributed to its phenolic nature, which acts by inhibiting certain early stages of the process, preventing the accumulation of toxic intermediate products formed in later stages [10, 77]. Melanin is also negatively charged and made up of polyphenolic chemicals and multifunctional polymers. Since phenolic chemicals are known to impede the synthesis of aflatoxin, they will prevent the buildup of harmful intermediates that are generated in the later steps of the route by inhibiting one or more early steps rather than late ones [78]. In the present study, the significant difference in binding energies (−9.5 vs −5.4 kcal/mol) between the two complexes suggests that melanin may have a stronger biological interaction with aflatoxin-producing *A. flavus* compared to ochratoxin-producing *A. carbonarius*. The presence of the specific hydrogen bond in the *A. flavus* complex likely contributes to this enhanced stability and could be a key factor in the molecular mechanism of interaction between these compounds. These findings provide valuable insights into the potential differential effects of melanin on different fungal toxin systems and could have implications for understanding fungal biology and potentially developing targeted interventions [79].

The molecular docking analysis reveals distinct mechanisms of action for melanin’s interaction with aflatoxin in *A. flavus* and ochratoxin in *A. carbonarius*. In *A. flavus*, melanin demonstrates a strong binding mechanism characterized by a significant binding energy of −9.5 kcal/mol. The primary mechanism involves a specific hydrogen-oxygen bond formation between Tyrosine 180 (TYR 180) and the melanin molecule, with a precise bond length of 2.20 Å. This strong hydrogen bonding suggests that melanin likely stabilizes the protein structure through direct interaction with the TYR 180 residue. The highly negative binding energy indicates a spontaneous and thermodynamically favorable interaction, suggesting that melanin could effectively modulate the protein’s function by maintaining a stable complex at the binding site. The presence of LEU 374 and other surrounding residues in the binding pocket appears to create a favorable microenvironment that enhances the stability of this interaction.

In contrast, the mechanism of action with ochratoxin in *A. carbonarius* follows a different pattern, characterized by a moderately strong binding energy of −5.4 kcal/mol. The absence of hydrogen bonds suggests that the interaction relies primarily on other forces, possibly including van der Waals interactions and hydrophobic effects. The binding pocket, formed by residues including VAL 130, ARG 129, GLU 36, HIS 44, ILE 178, and SER 173, creates a specific spatial arrangement that accommodates melanin through these non–hydrogen bonding interactions. The presence of both polar (ARG 129, GLU 36, HIS 44, SER 173) and nonpolar (VAL 130, ILE 178) residues in the binding site suggests a complex interaction mechanism involving both hydrophilic and hydrophobic regions of the protein. This similar with that reported in previous study about apposition of the aromatic rings ocratoxin and the indole nuclei of the melanin may also result in van der Waals’ forces, and the combination of these two types of forces may underlay the binding of ochratoxin to melanin [79]. The primary building block of melanin is an indole nucleus, and it is abundant in negatively charged groups such carboxyl groups and semi-quinones as well as its ionic interactions like van der Waals forces on the melanin polymer that is crucial for the affinity [80, 81].

Thus, the significant difference in binding energies and interaction mechanisms between the two systems suggests that melanin may have evolved different functional roles in these two fungal species. In *A. flavus*, the strong, specific hydrogen bonding mechanism indicates a potential regulatory role, possibly affecting aflatoxin production or metabolism. The weaker but still significant binding in *A. carbonarius*, mediated through non–hydrogen bonding interactions, suggests a more subtle modulatory effect on ochratoxin-related processes. These mechanistic insights could be particularly valuable for understanding how melanin might be used to differentially target these fungal species or their toxin production pathways. The stronger binding mechanism with aflatoxin-producing *A. flavus* suggests this might be a more promising target for melanin-based interventions.

Comparing the melanin-chelated heavy metal precipitate with the pure melanin using EDX and FTIR analyses found that fungal melanin exhibited significant concentration-dependent adsorption capacity. Also, the AAs analysis demonstrated that higher melanin concentrations result in greater removal efficiency of Cr6+ and Cd. The in vitro studies observed that the removal percentages of Cr and Cd were 60% and 77%, respectively, when the concentration of melanin was increased to 15 mg. Comparable results were reported by Nguyen et al. [52]. Additionally, the ability of melanin to bind various metal ions is one of the most prevalent characteristics of melanin pigments [82, 83]. As a potent and quick ion exchange molecule, melanin acts as a radical sink by binding pollutants, chemicals, and heavy metals. The significance of this characteristic in biology lies in the ability of melanin to chelate chemicals and control their absorption into cells [16]. In view of the functional groups elucidated by FTIR analysis, almost all of them were reported to chelate heavy metals by different mechanisms of action. Melanin, exhibited abundant functional groups such as=O, –OH, –NH, and –COOH through FTIR presented herein and that provides numerous binding sites for heavy metal ions [84]. Carboxylic groups elucidated in this study appeared also in a previous study to effectively adsorb heavy metals through coordination or chelation mechanisms, particularly cadmium [85]. Additionally, melanin binds predominantly to accessible carboxyl groups (acid base complex) as well as the free radicals produced by the comproportionating reactions were responsible for the complexation of metal ions on melanin pigment [85]. According to physicochemical analysis of fungal melanin which is generated from the precursor L-Dopa that appeared herein, should have quinone, semiquinone, carboxyl, amine, and hydroxyl (phenolic) groups by an almost similar mode of action [86]. Previous literature has showed that the number of active centers, accessibility, and affinity of the active centers for metal ions are some of the factors that determine the material’s capacity for chemical sorption [87]. Where, the adsorption of metals and its binding site is caused by distinct functional groups in melanin. For example, Pb2+ can bind to a variety of locations, such as carboxyl (COOH), amine (NH), and catechol (OH) groups. On the other hand, the carboxyl (COOH) group is the particular binding site for Cd2+, Zn2+, and Ca2+ [88]. Furthermore, the electronegativity may be the source of the variation in the binding affinities of the metal ions since the attraction to the negative charges of free radical intermediates plays a significant role in the binding of divalent metal ions. Finally, the order of metal electro- negativity is followed by the order of binding affinities for Cd and Cr according to different mechanisms of action that have hypothesized herein and have showed in the previous study [89].

### Conclusion

This study provides significant evidence regarding the bioremediation pipeline, enabling a natural fungal approach for melanin production and utilizing melanin as a heavy metal-chelating agent. In addition, the study demonstrated the antioxidant potential of melanin and its capability to inhibit the fungal growth and detoxification of mycotoxins aflatoxin B1 and ochratoxin A. This finding suggests potential applications for fungal melanin in eliminating heavy metals from water resources, removal of heavy metals fungal strains and preventing fungal and mycotoxin contamination in food. Furthermore, it will be necessary to explore its applications in industrial or agricultural settings.

## Electronic supplementary material

Below is the link to the electronic supplementary material.

Supplementary Material 1

## Data Availability

The genetic sequence of the strain analyzed has been submitted to the GenBank nucleotide sequence data- base at the National Library of Medicine, National Center for Biotechnology Information (NCBI). The assigned accession number for the sequence is MZ314535, which can be accessed at https://www.ncbi.nlm.nih.gov/nuccore/MZ314535.

## Abbreviations

*A. flavus*: *Aspergillus flavus*
*A. carbonarius*: *Aspergillus carbonarius*
DOPA: Dihydroxyphenylalanine
DPPH: 1,1-Diphenyl-2-picrylhydrazyl
DHN: Dihydroxynaphthalene
CD: Czapek–Dox
MS: Mineral salt
PD: Potato dextrose
AFB1: Aflatoxin B1
OTA: Ochratoxin
Cd: Cadmium
Cr: Chromium
FTIR: Fourier transform infrared
HPLC: High-performance liquid chromatography
EDX: Energy-dispersive X-ray
EC_50_: 50% of the DPPH radicals were scavenged
AAS: Atomic absorption spectrophotometer
LSD: Least significant difference

## Declarations

### Adherence to national and international regulations

All relevant institutional, national, and international guidelines and legislation were compiled or adhered to in the production of this study.
