# Supplementary material for: Suppression of mycotoxins production and efficient chelation of heavy metals using natural melanin originated from Aspergillus flavus and Aspergillus carbonarius
Source: BMC Biotechnol. 2025 Jan 11;25:6. doi: 10.1186/s12896-024-00941-7 (PMC11724575; doi:10.1186/s12896-024-00941-7)
Supplement: Supplementary file 1 — Supplementary Material 1 [file 12896_2024_941_MOESM1_ESM.docx]

| **Table S1:** Diagnostic tests for melanin identification produced by different filamentous fungi | | |
| --- | --- | --- |
| **Test** | **Producer fungal strain** | |
|  | ***A. flavus*** | ***A. carbonarius*** |
| **Solubility in water** | In Soluble | In Soluble |
| **Color** | Dark Brown | Black |
| **Solubility in KOH** | Soluble | Soluble |
| **Precipitation in 3N HCL** | Precipitated readily | Precipitated readily |
| **Solubility in organic acid solvent (chloroform, acetone, ethyl acetate)** | In Soluble | In Soluble |
| **Reaction with H_2_O_2_** | Decolorized | Decolorized |
| **Reaction with Fecl_3_**  **(Reaction for polyphenols test)** | Brown precipitate | Brown precipitate |
| **Reaction with sodium dithionite and potassium ferricyanide** | Decolorized and turned brown with addition of potassium ferricyanide | Decolorized and turned brown with addition of potassium ferricyanide |

| **Table S2:** FTIR for control melanin and melanin (15 mg) adsorption Cd | | | | |
| --- | --- | --- | --- | --- |
| **Peaks** | **Control** | **Sample** | **Shift** | **Functional group** |
| **1** | 3736 | 3785 | 49 | Alcohol group (O-H) stretch, free, strong and sharp |
| **2** | 3434 | 3408 | 26 | Alcohol group (O-H), stretch, H-bonded, strong and broad |
| **3** | 2924 | 2922 | 2 | Alkane group C-H (stretch, strong) |
| **4** |  | 2853 | - | Alkane group C-H (stretch, strong) |
| **5** | 2363 | 2361 | 2 | C≡ N stretching mode |
| **6** | 1632 | 1623 | 10 | C=C stretching mode, N-H bending in primary amine, or C=o stretching mode (amide) |
| **7** |  | 1710 | - | C=O stretching mode (Ketone)  Or C=O stretching mode (carboxylic acid) |
| **8** | 1456 | 1406 | 50 | C=C stretching mode (Aromatic ring) |
| **9** | 1260 | 1252 | 8 | C-O-H bending mode  C-O stretching mode (Alcohol)  C-O stretching mode (ether)  C=O bending mode  C-O carboxylic acid |
| **10** | 1147 | 1148 | 1 | C-O stretching mode (Alcohol)  C-O stretching mode (ether)  C-O stretching mode |
| **11** | 1028 | 1037 | 9 | C-O stretching mode (Alcohol)  C-O stretching mode (ether)  C-O stretching mode  C- F stretching mode |
| **12** | 928 |  | - | C=C bending, alkene, |
| **13** | 873 | 849 | 24 | Halo compound C-Cl, stretching |
| **14** | 815 |  | - | =C-H out of plane bending mode (Aromatic ring) or N-H, bending mode |
| **15** |  | 596 | - | C- Cl, C-Br, C-I (halo compound) |
| **16** | 541 | 542 | 1 | C- Cl, C-Br halo compound |
| **17** | 471 | 473 | 2 | C-Br, C-I (halo compound) |
| **18** |  | 447 | - | C-Br, C-I (halo compound) |

| **Table S3:** FTIR for control melanin and melanin (15 mg) adsorption Cr | | | |
| --- | --- | --- | --- |
| **Control** | **sample** | **Shift** | **Functional group** |
| 3736 | 3785 | 49 | Alcohol group (O-H) stretch, free, strong and sharp |
| 3434 | 3430 | 4 | Alcohol group (O-H), stretch, H-bonded, strong and broad |
| 2924 | 2923 | 1 | Alkane group C-H (stretch, strong) |
|  | 2853 | - | Alkane group C-H (stretch, strong) |
| 2363 | 2361 | 2 | C≡ N stretching mode |
|  | 1712 | - | C=O stretching mode (ketone)  C=O Stretching mode (carboxylic group ) |
| 1632 | 1626 | 6 | C=C stretching mode , N-H bending in primary amine , or C=o stretching mode (amide) |
| 1456 | 1405 | 51 | C=C stretching mode (Aromatic ring) |
| 1260 | 1258 | 2 | C-O-H bending mode  C-O stretching mode (Alcohol)  C-O stretching mode (ether)  C=O bending mode  C-O carboxylic acid |
| 1147 | 1151 | 4 | C-O stretching mode (Alcohol)  C-O stretching mode (ether)  C-O stretching mode |
|  | 1081 |  |  |
| 1028 | 1038 |  | C-O stretching mode (Alcohol)  C-O stretching mode (ether)  C-O stretching mode  C- F stretching mode |
| 928 |  |  | C=C bending , alkene, |
| 873 |  |  | Halo compound C-Cl , stretching |
| 815 |  |  | =C-H out of plane bending mode (Aromatic ring) or N-H, bending mode |
|  |  |  | C- Cl , C-Br , C-I ( halo compound ) |
| 541 |  | - | C- Cl, C-Br halo compound |
|  | 601 | - | C- Cl , C-Br , C-I ( halo compound |
|  | 538 | - | C-Br, C-I ( halo compound ) |
| 471 | 448 | 23 | C-Br, C-I ( halo compound ) |

**
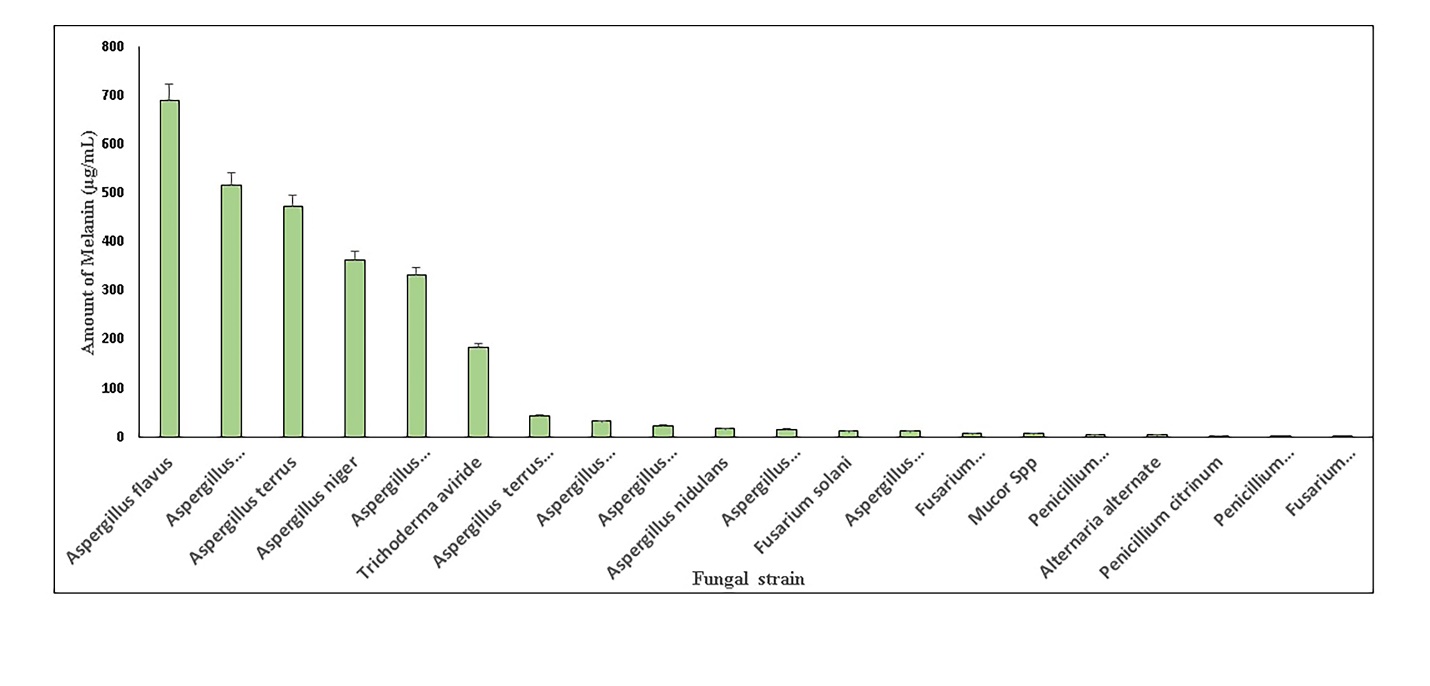
**

**Fig. S1 Melanin productivity by the isolated fungal strains. Data are presented as mean ±SEM from three independent experiments P ≤ 0.05.**


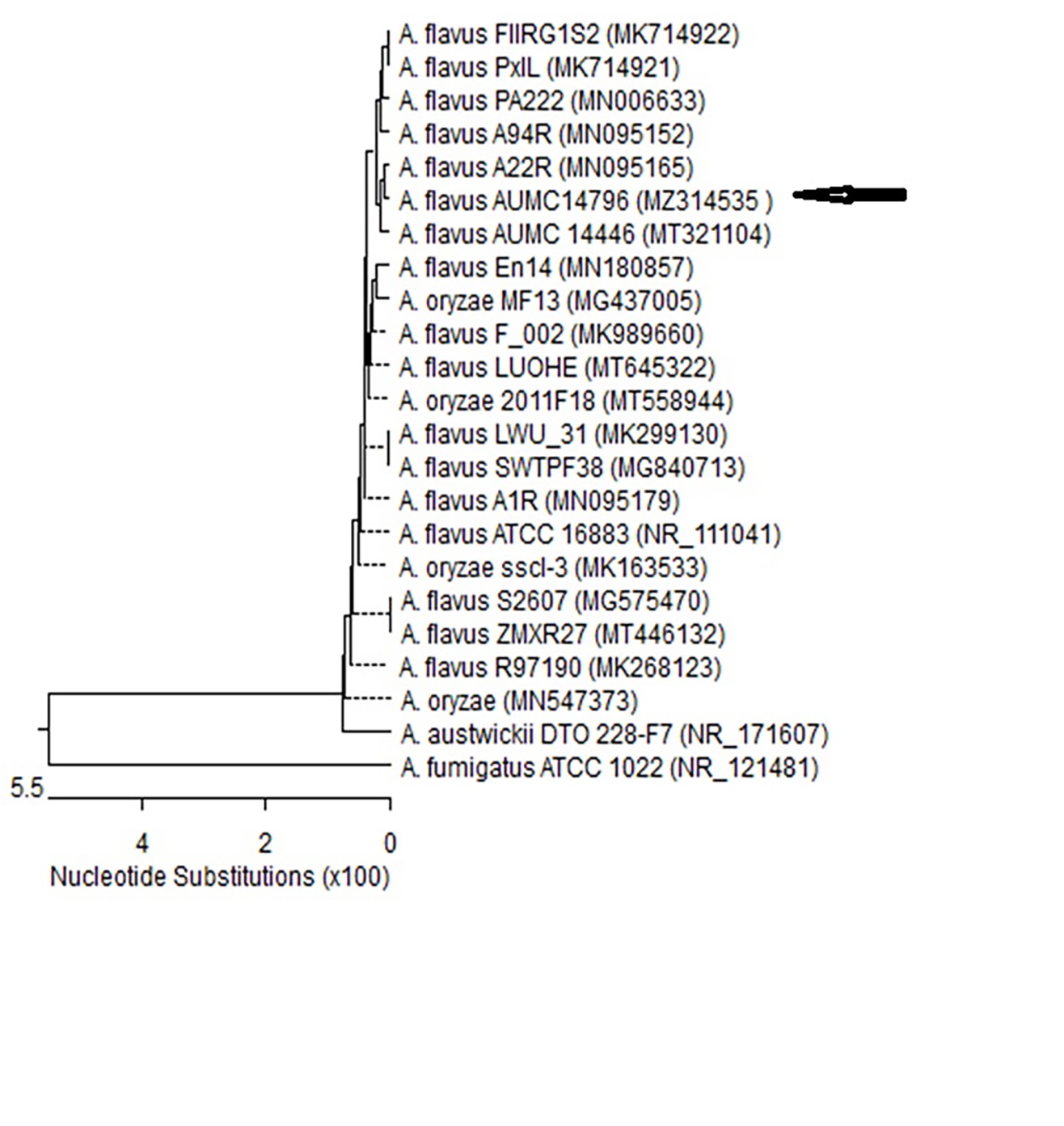


**Fig. S2 Phylogenetic tree based on ITS sequences of 18S rDNA of the fungal strain isolated in the present study (AUMC14796-MZ314535, arrowed) aligned with closely related sequences accessed from the GenBank. (A. = Aspergillus).**
